# Natural products targeting hepatitis B virus X protein and HBx-associated pathways in HBV-related hepatocellular carcinoma: mechanisms and therapeutic potential

**DOI:** 10.1080/13880209.2026.2724161

**Published:** 2026-09-04

**Authors:** Thu Ngoc Anh Nguyen, Piyanoot Thongsri, Yongyut Pewkliang, Mullika Traidej Chomnawang, Krit Thirapanmethee, Khanit Sa-ngiamsuntorn

**Affiliations:** a Department of Biochemistry, Faculty of Pharmacy, Mahidol University, Bangkok, Thailand; b Department of Pediatrics, Faculty of Medicine Ramathibodi Hospital, Mahidol University, Bangkok, Thailand; c Department of Microbiology, Faculty of Pharmacy, Mahidol University, Bangkok, Thailand; d Antimicrobial Resistance Interdisciplinary Center (AmRIC), Faculty of Pharmacy, Mahidol University, Bangkok, Thailand

**Keywords:** Hepatitis B virus, HBx protein, hepatocellular carcinoma, cccDNA, natural products, bioactive compounds, HBx‑targeted therapy

## Abstract

**Context:**

Hepatocellular carcinoma (HCC), largely driven by chronic hepatitis B virus (HBV) infection, remains a major global health challenge with limited curative options in advanced stages. The hepatitis B virus X protein (HBx) is a multifunctional viral oncoprotein that plays a central role in HBV persistence and hepatocarcinogenesis by disrupting transcriptional regulation, DNA repair, apoptosis, and immune responses.

**Objective:**

This review aims to evaluate the role of HBx in HBV‑related HCC progression and to examine the therapeutic potential of natural products that directly target HBx and indirectly modulate HBx‑driven antiviral and oncogenic pathways.

**Methods:**

A comprehensive literature analysis was conducted focusing on studies published over the past decade, including *in vitro*, *in vivo*, and clinical evidence addressing HBx-mediated mechanisms and the pharmacological effects of bioactive natural products, including plant‑derived phytochemicals and non‑plant natural compounds.

**Results:**

Accumulating evidence indicates that some natural products suppress HBV replication by reducing HBx expression, promoting HBx degradation, or limiting HBx‑dependent cccDNA transcription, whereas others primarily influence biological pathways associated with HBx‑driven hepatocarcinogenesis, including migration, invasion, fibrosis, metabolic reprogramming, and apoptosis resistance.

**Conclusion:**

Natural products represent a promising multi-target therapeutic strategy for HBV-related HCC by intervening in HBx-driven oncogenic processes. Future studies should focus on mechanistic validation, standardization of bioactive compounds, and well-designed clinical trials to support their translational application as adjunctive or alternative therapies.

## Introduction

Hepatocellular carcinoma (HCC) is the most prevalent type of primary liver cancer, accounting for over 90% of all liver malignancies, and ranks as the third leading cause of cancer-related mortality worldwide (Sung et al. 2021). HCC typically arises from hepatocytes and is strongly associated with chronic liver diseases, particularly viral hepatitis and cirrhosis (Motola-Kuba et al. 2006). Among viral infections, hepatitis B virus (HBV) plays a predominant etiological role in HCC, especially in endemic regions such as East and Southeast Asia and sub-Saharan Africa (Arbuthnot and Kew 2001; Llovet et al. 2016). From 2015 to 2030, HBV-related liver diseases including acute and chronic hepatitis, cirrhosis, and HCC are projected to cause over 20 million deaths globally, with HCC alone contributing to nearly 1 million of these fatalities (Yang et al. 2019).

HBV is a partially double-stranded DNA virus capable of integrating its genome into host chromosomal DNA, a feature that distinguishes it from other hepatotropic viruses such as hepatitis C virus (HCV), which does not integrate. This integration promotes persistent infection and long-term hepatic injury. HBV-mediated hepatocarcinogenesis involves multiple mechanisms, including genomic integration, chronic necroinflammation, and the oncogenic activities of HBV-encoded proteins, particularly the hepatitis B virus X protein (HBx) (Arbuthnot and Kew 2001; Yang et al. 2019). HBx, a small 154-amino acid regulatory protein, promotes viral transcription by regulating the epigenetic activity of the HBV cccDNA minichromosome and modulates multiple host cellular processes, including gene transcription, apoptosis, DNA repair, and signal transduction (Bouchard and Schneider 2004). These functions position HBx as a major driver of liver tumor initiation and progression.

Current first-line treatments for chronic HBV infection primarily include nucleos(t)ide analogs (NAs), such as entecavir, tenofovir disoproxil fumarate, and tenofovir alafenamide, as well as pegylated interferon-α (Peg-IFN-α), which effectively suppress viral replication but rarely eliminate the virus completely (Terrault et al. 2018; Yuen et al. 2018). A major limitation of these therapies is their inability to eliminate covalently closed circular DNA (cccDNA), the stable nuclear viral reservoir that serves as the transcriptional template for HBV replication and allows viral rebound after treatment discontinuation (Seeger and Mason 2015). In addition, these treatments do not fully restore antiviral immune responses, and patients receiving long-term antiviral therapy remain at risk of developing HCC (Revill et al. 2019). These limitations highlight the need for new therapeutic approaches targeting viral persistence and oncogenic drivers of HBV-associated hepatocarcinogenesis.

Natural products derived from plants, animals, and other biological sources, including traditional medicines, have attracted increasing attention for their dual antiviral and anticancer activities, particularly in the context of HBV-related HCC. These natural products possess various pharmacological properties, including suppression of viral replication, modulation of oxidative stress, regulation of inflammation, induction of apoptosis, and inhibition of tumor progression (Newman and Cragg 2020).

This review covers both direct HBx‑targeting natural products, which reduce HBx expression, stability, or interaction with cccDNA and host factors, and indirect HBx‑pathway modulators, which suppress HBx‑activated signaling pathways involved in viral replication, inflammation, metabolism, and hepatocarcinogenesis. Emphasis is placed on findings from the past decade, including both *in vitro* and *in vivo* studies, to elucidate the therapeutic potential of these natural agents in the prevention and treatment of HBV-associated liver cancer.

## Review methodology and literature selection

This narrative review was conducted to examine natural products targeting hepatitis B virus X protein (HBx) or HBx-associated pathways in hepatitis B virus (HBV) infection and HBV-related hepatocellular carcinoma (HCC). Literature searches were performed in PubMed, Scopus, Web of Science, and Google Scholar for studies published from January 2014 to December 2025, with a final literature update conducted in May 2026. Earlier studies published before 2014 were also considered when they provided mechanistic evidence that remained directly relevant to the objectives of this review. The following search strings were used, with minor database-specific formatting adjustments: (‘hepatitis B virus’ OR HBV) AND (HBx OR ‘hepatitis B virus X protein’) AND (‘natural product’ OR ‘natural compound’ OR phytochemical OR ‘bioactive compound’ OR herbal OR ‘traditional Chinese medicine’); (HBx OR ‘hepatitis B virus X protein’) AND (cccDNA OR ‘HBV replication’) AND (‘natural product’ OR phytochemical OR compound OR herbal); (HBx OR ‘hepatitis B virus X protein’) AND (‘natural product’ OR phytochemical OR compound); and (HBx OR ‘hepatitis B virus X protein’) AND (‘hepatocellular carcinoma’ OR HCC) AND (‘natural product’ OR phytochemical OR herbal OR compound).

Studies were included if they met the following criteria:

1. Investigated natural products or nature-derived compounds (plant, animal, or biologically derived);
2. Reported effects on HBx expression, function, or HBx-related biological processes relevant to HBV replication or HCC development;
3. Reported data from *in vitro*, *in vivo*, or clinical studies relevant to HBV infection or HCC;
4. Were original research articles or mechanistic studies published in peer-reviewed journals.

A total of 75 publications were screened by title and abstract, followed by full-text assessment for relevance to HBx, HBV replication, or HBV-related HCC. Following full-text evaluation, 18 primary mechanistic studies were included, comprising 10 studies in Tables 1 and 8 studies in Table 2. Review articles, conference abstracts, editorials, and studies lacking mechanistic relevance to HBx or HBx-associated pathways were excluded, although selected reviews were consulted for background information.

**Table 1.** List of natural compounds and products and their mechanism of action on HBx-related HBV infection.

Source	Compound	Source	Experimental model and IC_50_/ EC_50_/ Effective dosage	HBx-related mode of action	References
*Multi-herb formulas*					
Chinese herbal extract Su-duxing	Matrine, Oxymatrine, Chlorogenic acid, Sophocarpine, Baicalein, Wogonin	Crude extract	*In vitro* HepG2.2.15: IC_50_ 1.8 μg/mL, dosage 10 μg/mL; HepG2.A64: IC_50_ 2.1 μg/mL *In vivo* C57BL/6 mice: 45 mg/kg/day for 2 weeks	Possible reduction of HBx stability and viral replication relief. Suppress both wild-type and ETV-resistant HBV replication. Reduce HBsAg and HBeAg levels.	(Liu et al. 2018)
*Single compounds and single-herb interventions*					
*Prismatomeris connata*	Rubiadin	Isolation and purification from crude extract	*In vitro* HepG2.2.15: 4 μg/mL and 6 μg/mL	Down-regulate HBx protein expression. Inhibit secretion of HBV markers and HBV DNA replication.	(Peng et al. 2017)
*Microsorium fortunei*	Esculetin	Isolation and purification from crude extract	*In vitro* HepG2.2.15, HL-7702: 20–80 mg/L *In vivo* Ducklings: 20–80 mg/kg/day for 21 days	Inhibit the expression of HBx protein in a dose-dependent manner. Decrease DHBV DNA, DHBsAg, DHBeAg, ALT and AST.	(Huang et al. 2019)
*Melilotus officinalis*	Dicoumarol	Pure compound	*In vitro* HepG2, Huh-7: EC_50_ 100 μM for HiBiT-HBx; HepG2-NTCP: 100–300 μM; HepAD38: NR; PHHs: NR *In vivo* Alb-Cre transgenic mice C57BL/6-Tg [Alb-cre]21Mgn/J: 20 mg/kg/2 days for 21 days	Reduce HBx expression Reduce the recruitment of HBx to cccDNA and inhibit the transcriptional activity of cccDNA.	(Cheng et al. 2021)
*Heracleum laciniatum*	Sphondin	Pure compound	*In vitro* HepG2, HepG2-NTCP, Huh-7, HepAD38: 30 μM *In vivo* Alb-Cre transgenic mice C57BL/6-Tg [Alb-cre]21Mgn/J: 2.5 and 5 mg/kg/2 days for 16 days	Induce proteasomal degradation of HBx. Reduce the recruitment of HBx to cccDNA, inhibit cccDNA transcriptional activity and HBsAg expression.	(Ren et al. 2023)
*Centella asiatica*	Asiatic acid	Pure compound	*In vitro* HepG2-NTCP, Huh-7, PHHs: 10 µM, 20 µM and 30 µM; HepAD38: NR *In vivo* C57BL/6 mice: 30 mg/kg/2 days for 24 days	Promote the degradation of HBx through the autophagy-lysosomal pathway. Reduce the binding of HBx to cccDNA and inhibit cccDNA transcriptional activity.	(Li et al. 2024)
–	Furanocoumarin Fc-20 and Fc-31	Pure compounds	*In vitro* HEK293T, HepG2, HepG2.2.15: 50 μM for 24 h; HepG2-NTCP: IC_50_ 16.67 ± 1 μM for Fc-20 and 2.3 ± 0.23 μM for Fc-31	Increase proteasomal degradation of HBx. Disrupt the process of active histone modifications on cccDNA. Decrease HBV DNA, 3.5 kb RNA and cccDNA levels.	(Tyagi et al. 2024)
*Garcinia hanburyi*	Gambogic acid	Pure compound	*In vitro* HepG2, Huh-7, HepG2.2.15, HepAD38: NR HepG2-NTCP: 0.5 μM, 1 μM and 2 μM for 4 days *In vivo* C57BL/6 mice: 2.5 mg/kg/2 days for 20 days	Dose-dependent inhibition of HBx expression by upregulating the DTX1-Notch signaling pathway. Reduce extracellular HBV RNAs, HBV DNA, HBsAg, HBeAg and HBc protein.	(Wen et al. 2024)
*-*	All-*trans* retinoic acid	Pure compound	*In vitro* Hep3B, HepG2, Hep3B-NTCP, HepG2-NTCP: 1 μM for 24 h	Induce ubiquitination and proteasomal degradation of HBx by up-regulating p53 and Siah-1 levels.	(Han and Jang 2023)
			*In vitro* Hep3B, HepG2, Hep3B-NTCP, HepG2-NTCP: 5 μM for 24 h	Induce E6-associated protein (E6AP)-mediated proteasomal degradation of HBx to suppress HBV replication in a p53-dependent pathway	(Han and Jang 2024)

NR: not reported or not clearly specified in the cited study; IC_50_: half-maximal inhibitory concentration; EC_50_: half-maximal effective concentration. When IC_50_/EC_50_ values were not available, tested concentrations or in vivo doses are shown as reported in the original study.

**Table 2.** Natural compounds and products targeting HBx-associated pathways in hepatocellular carcinoma and their proposed mechanisms of action.

Source	Compound	Source	Experimental model and IC_50_/ EC_50_/ Effective dosage	HBx-related mode of action	References
*Multi-herb formulas*					
Chinese medicine Sini-San	–	Crude extract	*In vitro* HepG2: 100, 200, 400 and 800 μg/ml	Suppress MMP-9 transcription by inhibiting AP-1 and NF-κB activity through suppression of HBx-induced activation of Ras/Raf/ERK, JNK, and PI3K/AKT/PKB signaling pathways.	(Lin et al. 2015)
*Compound Phyllanthus urinaria L*	Gallic acid, quercetin, and luteolin	Crude extract	*In vitro* HepG2-HBx: IC_50_ 144.2 μg/ml, dosage 70 *μ*g/ml and 35 *μ*g/ml *In vivo* BABL/c nude mice: 25 mg/kg and 300 mg/kg	Inhibit the mRNA and protein expression of HBx and HBx-activated SHH, PTCH-1, and GLI-1 signaling.	(Li et al. 2019)
*Single compounds and single-herb interventions*					
–	Ursolic acid	Pure compound	*In vitro* Huh-7: NR	Suppress HBx-mediated RhoA activation, beclin-1 promoter activation and subsequent autophagy induction. Reverse HBx-induced anti-cancer drug resistance.	(Chang et al. 2016)
			*In vitro* Huh-7, HepG2, Hep3B: 9 μM *In vivo* Nu/Nu mice: 50 mg/kg/day for 8 weeks	Suppresses HBx-induced Sp-1 and Smad3/4 activation, blocks MMP-3 secretion and hepatoma cell migration, and restores TGF-β-induced apoptosis by reversing HBx survival signaling.	(Wu et al. 2011)
–	Naringenin	Pure compound	*In vitro* HepG2, HepG2-HBx: 25, 50, and 100 μM *In vivo* Male mice of the line A106 in the C57BL/6 background: 30 mg/kg/day p.o. for 14 days	Inhibit HBx-induced expression of hepatic adipogenic and lipogenic genes SREBP1c, LXRα, and PPARγ.	(Lin et al. 2017)
*Rheum palmatum* L	Chrysophanol	Pure compound	*In vitro* HSC-T6: NR	Impair HBx-induced activation of hepatic stellate cells (HSCs) *via* ER stress, ferroptosis-dependent and GPX4-independent pathways.	(Kuo et al. 2020)
			*In vitro* HSC-T6, LX-2 human HSC line: 30 μM *In vivo* C57B/6 female mice: 10 mg/kg, 3 times per week for 2 weeks	Inhibition of HBx-modulated mTOR, PPARs, α-SMA, CTGF signaling pathways and decrease HSC activation and liver fibrosis.	(Lin et al. 2024)
*Bufo Bufo gargarizans*	Bufalin	Pure compound	*In vitro* HepG2.2.15: IC_50_ 3.2 nM; PLC5: IC_50_ 3.6 nM; Huh7: IC_50_ 15 nM; HepG2: IC_50_ 20 nM; HLF: NR; LO2: NR; dosage 10 nM *In vivo* Female athymic nude mice: 20 μg/kg, 3 times per week for 5 weeks	Inhibition of HBx-induced AR/CCRK/β-catenin signaling by targeting AR and CCRK, leading to cell cycle arrest and HCC regression	(Yu et al. 2020)

NR: not reported or not clearly specified in the cited study; IC_50_: half-maximal inhibitory concentration; EC_50_: half-maximal effective concentration. When IC_50_/EC_50_ values were not available, tested concentrations or *in vivo* doses are shown as reported in the original study.

For Table 3, additional literature was screened using the compounds identified in Tables 1 and 2 as search terms combined with keywords including ‘pharmacokinetics’, ‘bioavailability’, ‘toxicity’, ‘safety’, ‘clinical’, ‘formulation’, and ‘delivery system’. Studies were included if they provided information relevant to safety, pharmacokinetics, translational feasibility, or clinical development, regardless of disease indication.

**Table 3.** Safety, pharmacokinetics, translational evidence, advantages and limitations.

Compound/Extract	Safety profile	PK/Bioavailability	Translation status	Advantages	Limitations	References
Rubiadin	Limited toxicology	Poorly characterized; likely low oral bioavailability	Early preclinical studies, no documented animal PK, toxicology or HBV-HCC PK/safety studies.	Reduces HBx expression and HBV markers.	Lack of *in vivo* validation; unknown exposure and safety in liver disease.	(Rao et al. 2006; Peng et al. 2017)
Su-duxing	Acceptable in HBV mouse models	Multi‑ component, unclear systemic exposure	Traditional clinical use and preclinical experimental studies. HBV-relevant preclinical evidence.	Suppresses wild type and resistant HBV, synergizes with entecavir.	Limited PK data; no clinical trials in HBV‑HCC populations.	(Liu et al. 2018)
Esculetin	Low toxicity	Moderate absorption, rapid metabolism	Preclinical evidence, no human PK data. Mostly non-HBV evidence.	Antiviral and hepatoprotective activity.	No human clinical trials evaluating efficacy in HBV or HCC.	
Dicoumarol	Bleeding risk due to anticoagulant activity	Good oral bioavailability	Historically approved and clinically used as an oral anticoagulant. Mainly from anticoagulant and non-HBV studies.	Clear HBx degradation and cccDNA silencing.	Anticoagulant liability; not suitable for chronic HBV HCC.	(Sun et al. 2020; Cheng et al. 2021; Yang et al. 2025)
Sphondin	Low toxicity *in vivo*	Limited PK data	HBV-relevant preclinical evidence.	Direct HBx binding; strong HBsAg reduction; NAs synergy.	Solubility and exposure are not defined.	(Yang et al. 2002; Ren et al. 2023)
Fc-20 and Fc-31	Toxicity not assessed *in vivo*	No PK data	*In vitro* only (advanced HBV systems)	Potent direct HBx degradation. Defined chemical profile enabling optimization.	No *in vivo* validation or safety data available.	(Bruni et al. 2019; Tyagi et al. 2024)
Asiatic acid	Low to moderate toxicity	Poor solubility, liver accumulation	Advanced preclinical stage. PK/safety data mainly from non-HBV studies.	Epigenetic silencing of cccDNA; hepatoprotective.	Poor solubility and low oral bioavailability Lack of human PK data.	(Grimaldi et al. 1990; Li et al. 2024; Dong et al. 2025; Liang et al. 2025)
Gambogic acid	Narrow therapeutic window	Very poor oral PK; IV preferred	Late preclinical to early clinical stage testing for cancer indications. No HBV-specific safety studies.	Strong HBx suppression and antiviral activity. Early clinical exposure *via* intravenous formulations.	Extremely poor oral bioavailability. Systemic toxicity. No clinical trials in HBV or HCC.	(Hatami et al. 2020; Wen et al. 2024; Xu et al. 2025)
All-*trans* retinoic acid	Hepatic toxicity reported; limits use in chronic HBV	Well‑defined human PK	Fully clinically validated drug. Preclinical evidence in liver disease. Clinical PK established outside HBV-related HCC.	Dual HBx degradation mechanisms.	Dose‑limiting toxicities in liver disease. Limited clinical studies in chronic liver disease contexts.	(Roenigk Jr 1988; Adamson 1994; Woods and Norsworthy 2023)
Sini-San	Long clinical safe use in liver disorders	Multi- component, low to moderate oral bioavailability, gut microbiota-mediated metabolism.	Extensive historical and modern clinical use for chronic liver diseases. Limited HBV-HCC-specific evaluation.	Suppresses HBx-driven migration and invasion.	Indirect action; no effect on HBV replication. Lack of randomized clinical trials in HBV‑HCC.	(Jiang et al. 2003; Lin et al. 2015; Li et al. 2026)
Ursolic acid	Generally safe	Poor solubility, low oral PK	Advanced preclinical stage for anti‑HCC effects.	Broad anticancer activity. Reverses HBx-mediated chemoresistance.	Poor oral absorption and rapid metabolism. Lack of clinical trials in HBV‑related HCC.	(Wu et al. 2011; Wang et al. 2013; Zhu et al. 2013; Chang et al. 2016; Panda et al. 2022)
Naringenin	Excellent human safety	Human oral PK available	Human phase‑I study showed safe pharmacokinetics. Preclinical liver disease and HCC models.	Metabolic effects relevant to HBx‑driven carcinogenesis.	More effective for prevention or early‑stage disease rather than advanced HCC. Limited cancer‑specific clinical trials.	(Lin et al. 2017; Joshi et al. 2018; Rebello et al. 2020)
Compound *Phyllanthus urinaria L*	Widely safe in chronic use	Extract- dependent PK	Human clinical trial data in chronic HBV.	Human clinical trial evidence demonstrates safety in chronic HBV infection, antiviral and hepatoprotective effects.	Limited efficacy as monotherapy. Unclear optimal dosing.	(Chan et al. 2003; Jung et al. 2015; Li et al. 2019; Liu et al., 2024)
Chrysophanol	Hepatic toxicity reported with repeated dosing	Rapid metabolism	Preclinical stage with *in vitro* and animal studies. Mainly non-HBV/HCC toxicology and pharmacology studies.	Demonstrates anti‑proliferative and metabolic regulatory effects. Targets HBx driven fibrotic mechanisms.	Clear hepatotoxicity and nephrotoxicity with repeated dosing, unsuitable for long-term use.	(Xie et al. 2019; Kuo et al. 2020; Su et al. 2020; Lin et al. 2024)
Bufalin	Cardiotoxic risk	Rapid clearance	Limited clinical through traditional medicine and experimental oncology settings. Limited HBV/HCC-specific safety evidence.	Extremely potent anticancer activity at low concentrations. Potent inhibition of HBx-mediated AR/β-catenin signaling.	Severe cardiotoxicity, unsuitable for chronic treatment.	(Botelho et al. 2019; Yu et al. 2020; Gao et al. 2025)

## Hepatocellular carcinoma characteristics

Hepatocellular carcinoma (HCC) is a primary malignant tumor of the liver that typically arises in the context of chronic liver disease and cirrhosis (Llovet et al. 2016). The principal risk factors for HCC include chronic hepatitis B virus (HBV) and hepatitis C virus (HCV) infections, excessive alcohol consumption, exposure to dietary aflatoxins, and metabolic disorders such as nonalcoholic fatty liver disease (NAFLD) and diabetes mellitus. While the prevalence of these risk factors varies geographically, chronic viral hepatitis remains the leading cause of HCC in many endemic regions (Gomaa et al. 2008).

**Figure 1.**
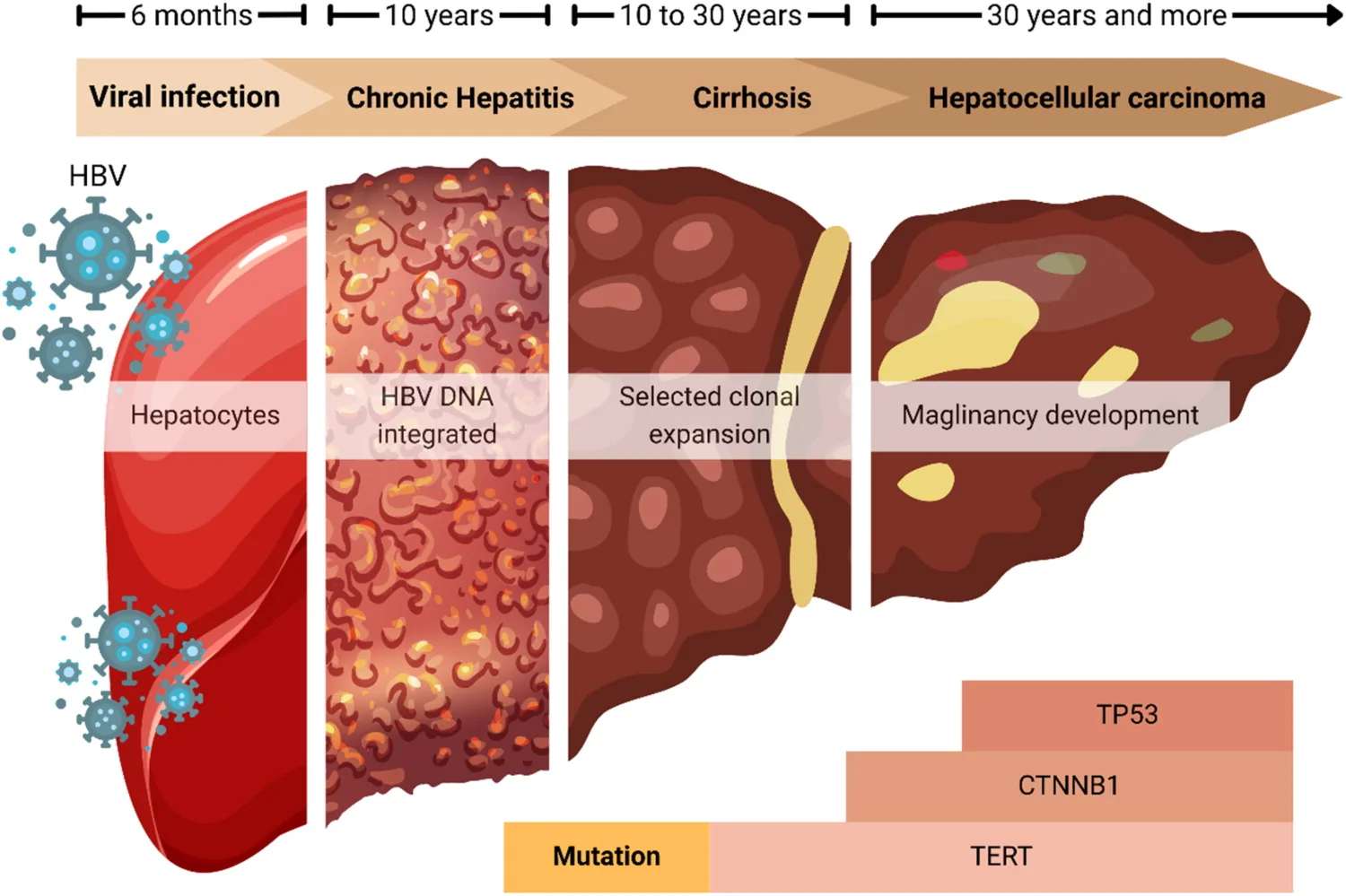
Progression of HBV infection to hepatocellular carcinoma and associated genetic mutations. (Create by BioRender.com / Mahidol University).

The development of HCC is a multistep process characterized by persistent hepatic inflammation, regenerative hyperplasia, and accumulation of genetic and epigenetic alterations (Llovet et al. 2022). Chronic liver injury results in repeated cycles of hepatocyte damage and regeneration, increasing the probability of mutations and malignant transformation. Key molecular signaling pathways frequently implicated in HCC pathogenesis include the Wnt/β-catenin, phosphoinositide 3-kinase (PI3K)/Akt/mammalian target of rapamycin (mTOR), and mitogen-activated protein kinase (MAPK)/extracellular signal-regulated kinase (ERK) pathways. Genetic alterations such as mutations in telomerase reverse transcriptase (TERT), tumor protein p53 (TP53), and catenin beta-1 (CTNNB1) are commonly observed in HCC tissues (Vij and Calderaro 2021) (Figure 1).

## Hepatitis B virus biology and life cycle

**Figure 2.**
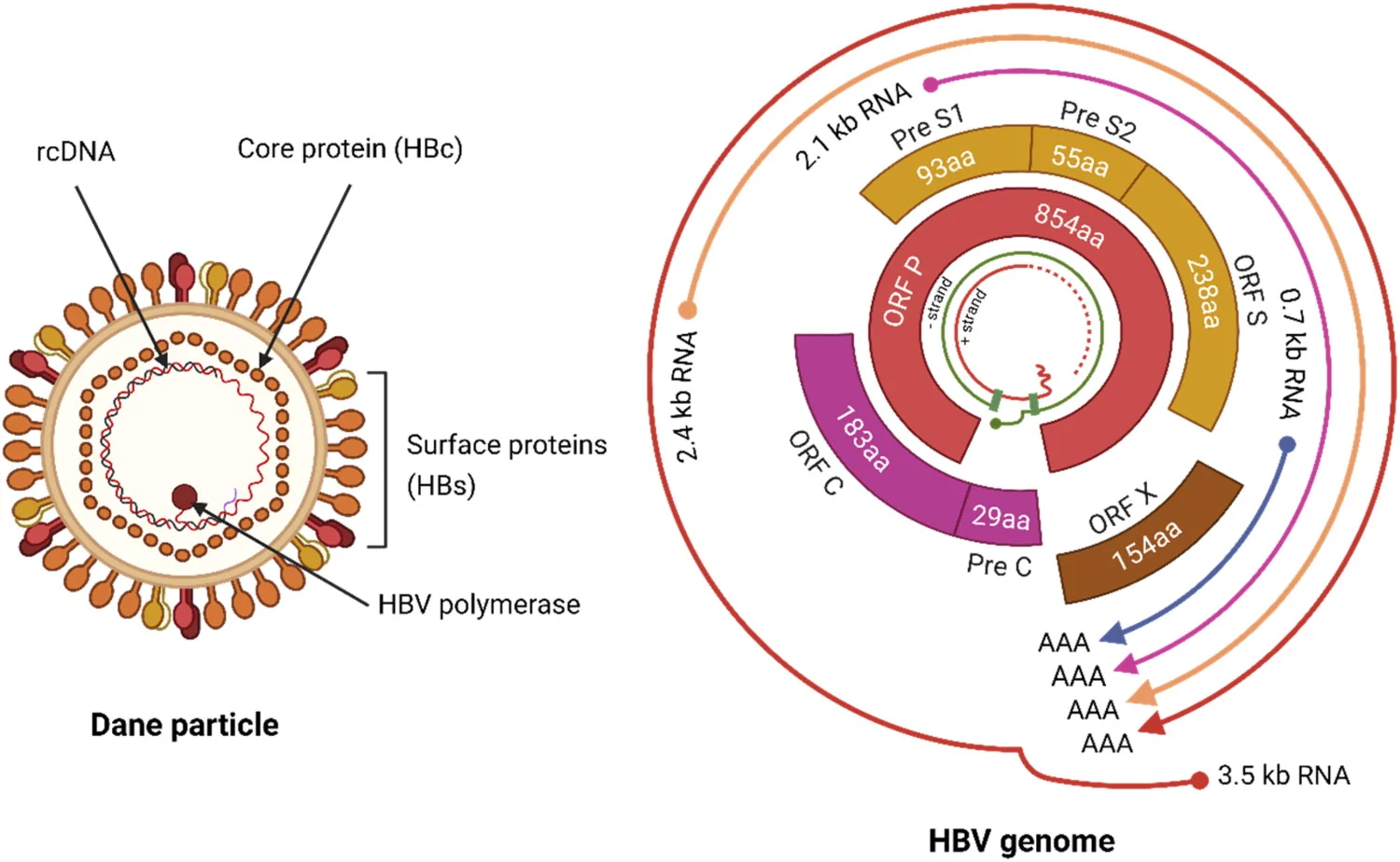
Schematic representation of HBV particles and HBV genome. Left, infectious HBV virion (dane particle). Right, the encoded open reading frames (C, P, S, X), RNA transcripts, and genomic organization of the hepatitis B virus. (Create by BioRender.com / Mahidol University).

Hepatitis B virus (HBV) is a partially double-stranded DNA virus classified within the *Orthohepadnavirus* genus of the *Hepadnaviridae* family (IARC Working Group on the Evaluation of Carcinogenic Risks to Humans 2012). HBV virions, also known as Dane particles, are spherical and enveloped, with a diameter of approximately 42 nm. The outer lipid envelope contains hepatitis B surface antigens (HBsAg), which are classified into small (S), medium (M), and large (L) glycoprotein forms (IARC Working Group on the Evaluation of Carcinogenic Risks to Humans 2012). Beneath the envelope, the nucleocapsid is composed of hepatitis B core antigen (HBcAg), which encases the viral genome and a reverse transcriptase/DNA polymerase enzyme (IARC Working Group on the Evaluation of Carcinogenic Risks to Humans 2012; Tsukuda and Watashi 2020). The HBV genome is approximately 3.2 kilobases in length and consists of four overlapping open reading frames (ORFs): pre-S/S (encodes HBsAg), pre-C/C (encodes HBcAg and HBeAg), P (polymerase), and X (encodes the HBx protein) (Figure 2)  (Tsukuda and Watashi 2020). HBx is a multifunctional regulatory protein essential for efficient viral replication and pathogenesis of HBV-associated liver diseases (Bouchard and Schneider 2004).

The HBV life cycle comprises several stages: attachment, entry, uncoating, nuclear import, transcription, translation, reverse transcription, and virion assembly and release (Seeger and Mason 2015; Tsukuda and Watashi 2020). Infection begins with the attachment of HBV to heparan sulfate proteoglycans on hepatocyte surfaces, followed by specific binding of the viral L-HBsAg to the sodium taurocholate cotransporting polypeptide (NTCP), a bile acid transporter that mediates viral entry *via* endocytosis (IARC Working Group on the Evaluation of Carcinogenic Risks to Humans 2012; Seeger and Mason 2015). Upon entry, the nucleocapsid is transported to the nucleus, where relaxed circular DNA (rcDNA) is released and converted by host DNA repair enzymes into covalently closed circular DNA (cccDNA), a stable episomal form that serves as the transcriptional template for viral mRNAs (Seeger and Mason 2015; Tsukuda and Watashi 2020). Transcription of cccDNA is mediated by host RNA polymerase II and controlled by four distinct viral promoters. This process produces several viral transcripts: the 3.5 kb pregenomic RNA (pgRNA), precore mRNA, 2.4 kb preS1 mRNA, 2.1 kb preS2/S mRNA, and 0.7 kb X mRNA (Beck and Nassal 2007; Tsukuda and Watashi 2020). These transcripts are translated in the cytoplasm to produce viral structural and regulatory proteins, including HBsAg, HBcAg, HBeAg, polymerase, and HBx  (Tsukuda and Watashi 2020). The pgRNA, in particular, serves both as the mRNA for core and polymerase and as the template for reverse transcription during genome replication (Beck and Nassal 2007). Inside newly formed nucleocapsids, pgRNA undergoes reverse transcription by the viral polymerase, resulting in the formation of negative-strand DNA, followed by partial synthesis of the positive strand to generate rcDNA  (Seeger and Mason 2015; Tsukuda and Watashi 2020). Mature nucleocapsids can either recycle to the nucleus to maintain or amplify cccDNA levels, or be enveloped by HBsAg-containing membranes in the endoplasmic reticulum and secreted as progeny virions  (Vij and Calderaro 2021). In addition to infectious virions, HBV-infected cells also release large quantities of noninfectious subviral particles composed solely of HBsAg, which contribute to immune tolerance and evasion  (Tsukuda and Watashi 2020).

**Figure 3.**
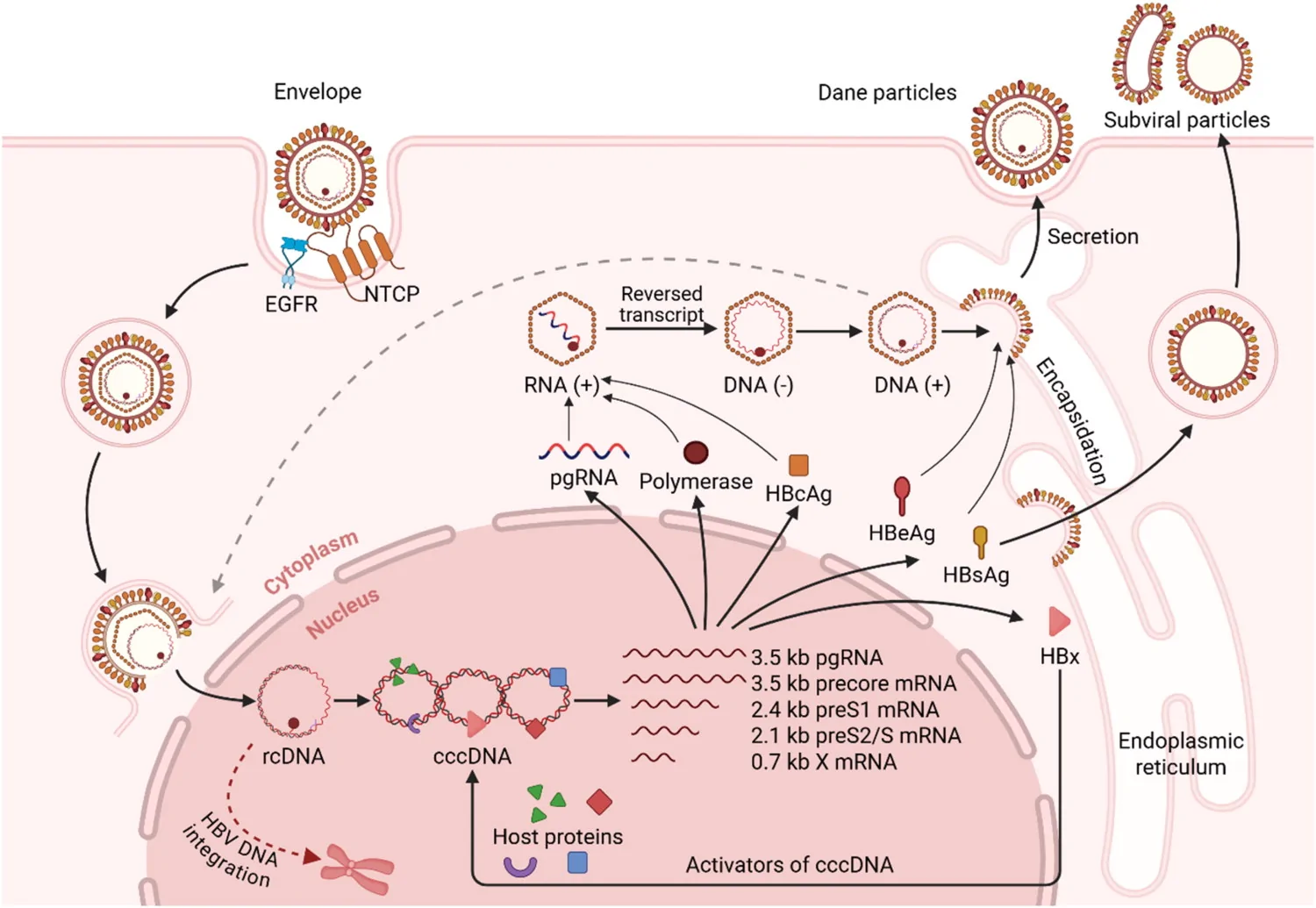
Schematic overview of the hepatitis B virus (HBV) life cycle. Refer to the main text for detailed explanation. (Create by BioRender.com / Mahidol University).

HBV establishes chronic infection primarily by maintaining the nuclear cccDNA reservoir, which is resistant to current antiviral therapies (Figure 3) (Seeger and Mason 2015).

## Molecular characteristics of HBx protein and role of HBx in HBV persistence and development of HCC

The hepatitis B virus X protein (HBx) is a multifunctional, nonstructural regulatory protein encoded by the X open reading frame. Comprising 154 amino acids with a molecular mass of approximately 17 kDa, HBx is highly conserved among mammalian *Hepadnaviridae* and plays a pivotal role in both HBV replication and the pathogenesis of HBV-related liver diseases, including chronic hepatitis, cirrhosis, and hepatocellular carcinoma (HCC) (Bouchard and Schneider 2004).

**Figure 4.**
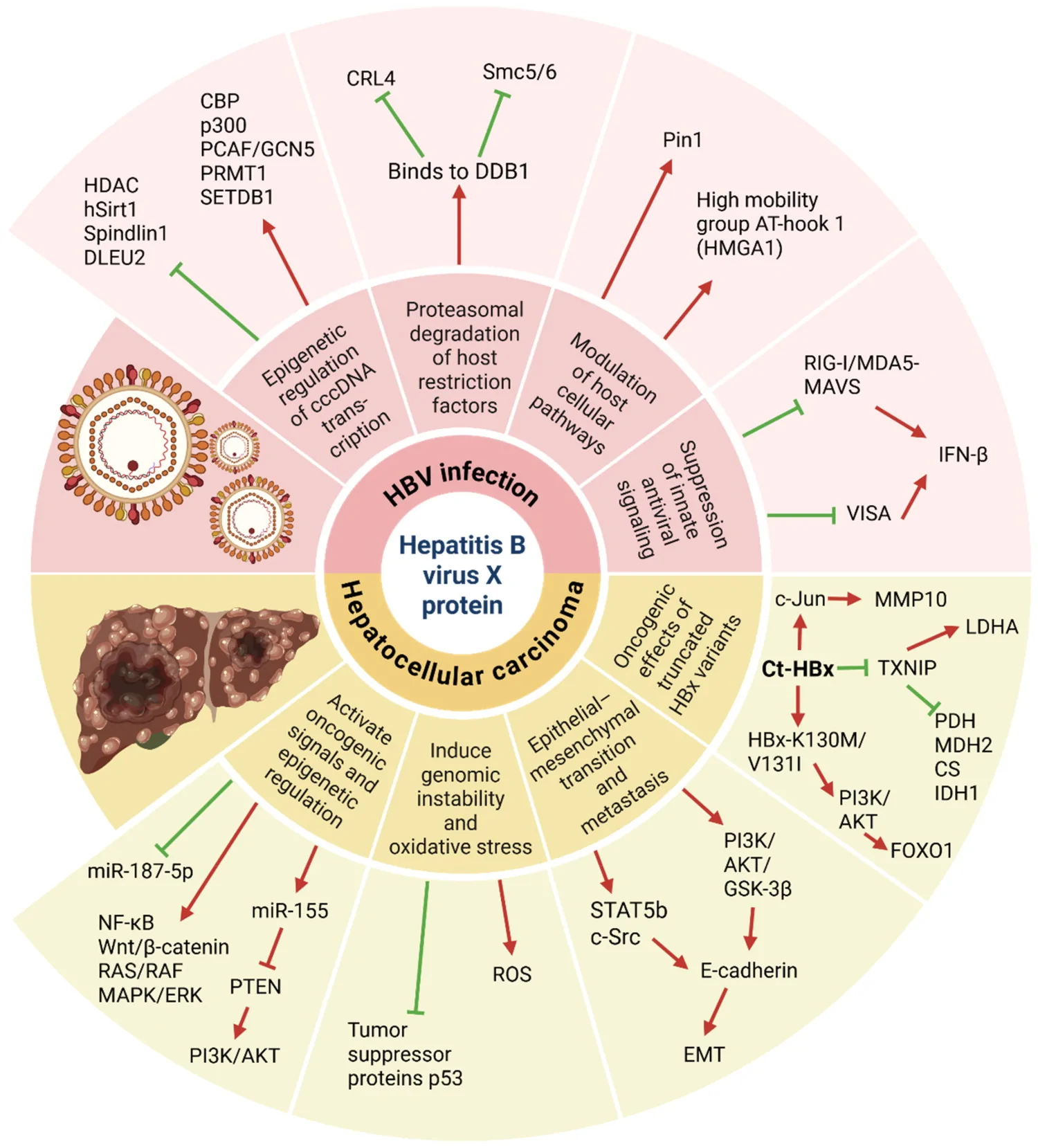
Molecular hallmarks of HBV infection and hepatocellular carcinoma modulated by hepatitis B virus X protein (HBx). (Create by BioRender.com / Mahidol University).

HBx exerts its functions through interactions with host transcriptional regulators, epigenetic modifiers, and signal transduction molecules. It is increasingly recognized as a central molecular target for therapeutic intervention due to its dual roles in sustaining HBV persistence and promoting oncogenic transformation of hepatocytes (Figure 4) (Slagle and Bouchard 2018; Sivasudhan et al. 2022).

## Role of HBx in HBV replication and persistence

### Epigenetic regulation of cccDNA transcription

One of the primary roles of HBx is to enhance HBV transcription and replication. Upon synthesis in the cytoplasm, HBx protein returns to the nucleus where it binds to the HBV cccDNA minichromosome and modulates the recruitment of host epigenetic modifiers. HBx promotes the binding of histone acetyltransferases, including CREB-binding protein (CBP), E1A-binding protein p300 (p300), p300/CBP-associated factor (PCAF; also known as lysine acetyltransferase 2B, KAT2B), and general control nonderepressible 5 (GCN5; also known as lysine acetyltransferase 2A, KAT2A), while reducing the repressive activity of histone deacetylase 1 (HDAC1) and sirtuin 1 (SIRT1). This shift increases acetylation of cccDNA-bound histones and promotes transcriptional activation of the viral minichromosome (Belloni et al. 2009; Kim et al. 2016; Slagle and Bouchard 2018).

HBx also interacts with Spindlin1, a chromatin reader protein, and inhibits transcriptional suppressors, including protein arginine methyltransferase 1 (PRMT1), and histone methyltransferase SETDB1 to enhance recognition of specific histone marks, leading to an epigenetic switch from a repressive state, marked by histone H3 lysine 9 di- and trimethylation (H3K9me2/3), to an active state, marked by H3K4me3, on the cccDNA minichromosome, resulting in reducing transcriptional silencing and promoting viral replication (Benhenda et al. 2013; Rivière et al. 2015; Liu et al. 2023).

Recent research suggests that HBx interacts directly with the promoter region of long noncoding RNA deleted in lymphocytic leukemia 2 (DLEU2), enhancing its transcription and leading to the accumulation of DLEU2 transcripts in infected hepatocytes. Once upregulated, DLEU2 physically interacts with HBx and the histone methyltransferase enhancer of zeste homolog 2 (EZH2), a component of the polycomb repressor complex 2 (PRC2). On viral cccDNA, HBx–DLEU2 complexes displace EZH2, relieving repressive H3K27me3 marks and thereby promoting active transcription of HBV genes (Salerno et al. 2020).

### Proteasomal degradation of host restriction factors

HBx facilitates viral replication by targeting host restriction factors for degradation through the ubiquitin–proteasome system. A key interaction occurs between HBx and damaged DNA-binding protein 1 (DDB1), an adapter protein of the Cullin 4-RING E3 ubiquitin ligase (CRL4) complex. Through this interaction, HBx hijacks the CRL4 ubiquitin ligase machinery to promote ubiquitination and proteasomal degradation of specific host proteins (Hodgson et al. 2012; Slagle and Bouchard 2018).

Notably, the structural maintenance of chromosomes 5 and 6 complex (Smc5/6) has been identified as a restriction factor targeted by the HBx-DDB1 interaction (He et al. 2023). By promoting the degradation of Smc5/6, HBx decreases transcriptional repression of the viral cccDNA, thereby promoting a cellular environment conducive to viral replication (Leupin et al. 2005; Minor et al. 2020).

### Modulation of host cellular pathways supporting viral replication

HBx also relies on host cellular proteins to maintain efficient viral replication. HBx requires Pin1, a peptidyl-prolyl cis/trans isomerase NIMA-interacting 1, to restore replication in HBx-deficient HBV (Zhou et al. 2021). Inhibiting Pin1 expression in HepG2.2.15 cells lead to a significant decrease in the levels of HBsAg and HBeAg and blocks HBx’s ability to rescue viral replication (Zhou et al. 2021).

Moreover, a study revealed that high mobility group AT-hook 1 (HMGA1) functions as a positive regulator of HBV replication through a reciprocal relationship with the viral HBx protein. HMGA1 directly binds to HBV cccDNA and enhances its transcriptional activity, thereby increasing viral RNA synthesis and replication. At the same time, HBx upregulates HMGA1 expression in host cells, forming a reinforcing loop in which HMGA1 promotes HBV replication while HBx further boosts HMGA1 levels (Shen et al. 2022).

### Suppression of innate antiviral signaling

HBx has been shown to undermine innate antiviral signaling by destabilizing key host proteins involved in type I interferon (IFN‐I) induction. HBx interacts with MAVS (mitochondrial antiviral signaling protein), thereby impeding the RIG-I/MDA5-MAVS pathway and reducing IFN-β production (Wang et al. 2010). Additional studies found that HBx suppresses signaling downstream of virus sensors such as VISA (also called MAVS/IPS-1 in some contexts), again lowering IRF3 activation and IFN expression (Kumar et al. 2011). By attenuating this signaling, HBx effectively removes the IFN-I–dependent brake that normally restricts HBV replication, allowing the viral life cycle to proceed unchecked (Kuipery et al. 2020).

## Regulation of HBx in the development of HCC

### Activation of oncogenic signaling pathways and epigenetic regulation

HBx is a multifunctional oncogenic driver in HCC development, primarily through its interactions with host cellular pathways and contribution to chronic inflammation, genomic instability, and epigenetic regulation (Sivasudhan et al. 2022). HBx induces epigenetic modifications by modulating non-coding RNAs, such as downregulating miR-187-5p and upregulating miR-155, to promote proliferation, migration, and invasion in HCC cells (Niu et al. 2022; Deng et al. 2023). By suppressing PTEN *via* miR-155, HBx activates the PI3K/AKT pathway, enhancing cell survival and metastatic potential (Niu et al. 2022). Additionally, HBx contributes to HCC through the activation of oncogenic signaling pathways including NF-κB, Wnt/β-catenin, RAS/RAF, and MAPK/ERK pathways, which are involved in cell proliferation, disrupting cell cycle regulation, promoting inflammation and resistance to apoptosis (Zhou et al. 2022; Agustiningsih et al. 2024).

### Induction of genomic instability and oxidative stress

HBx promotes genomic instability, a hallmark of cancer development, by impairing DNA damage response and repair mechanisms (Sivasudhan et al. 2022). HBx interacts with and functionally inhibits tumor suppressor proteins such as p53, compromising cell‑cycle checkpoint control and allowing damaged hepatocytes to evade apoptosis (Elmore et al. 1997). Moreover, HBx directly generates reactive oxygen species (ROS), inducing oxidative stress in hepatic cells which causes DNA damage and chromosomal aberrations that further drive carcinogenesis (Wu et al. 2013; Li et al. 2022).

### Promotion of epithelial-mesenchymal transition and metastasis

Another important aspect of HBx function is its ability to drive metastasis and invasion of hepatoma cells. HBx enhances the metastatic and invasive potential of hepatoma cells by promoting epithelial-mesenchymal transition (EMT), a process marked by loss of epithelial characteristics and acquisition of mesenchymal traits. Mechanistically, HBx activates signaling pathways involving STAT5b and c‑Src, leading to repression of E‑cadherin, a key cell-cell adhesion molecule, and induction of mesenchymal markers such as vimentin and fibronectin (Lee et al. 2006; Yang et al. 2009). HBx also stabilizes Snail protein, a key EMT driver, by activating the PI3K/AKT/GSK-3β pathway. Phosphorylation of GSK-3β by AKT inhibits its ability to degrade Snail, leading to Snail accumulation, which suppresses E-cadherin and promotes mesenchymal traits, enhancing cell migration and invasion *in vitro* and *in vivo* (Liu et al. 2012).

### Oncogenic effects of truncated HBx variants

Truncated forms of HBx, particularly C-terminal mutants that arise after HBV DNA integrates into the host genome, appear to amplify oncogenic potential. The C-terminal truncated HBx (ct-HBx) proteins lose the pro-apoptotic properties of the full-length HBx, which can enhance properties such as cell invasiveness, metastasis, and activation of pro-oncogenic transcription factors like c-Jun protein which in turn increases the transcription of matrix metalloproteinase 10 (MMP10) (Sze et al. 2013). Ct-HBx variants HBx-120 and HBx-134 alter cellular metabolism in HCC by inducing a metabolic shift from mitochondrial oxidative phosphorylation to aerobic glycolysis, commonly known as the Warburg effect. Mechanistically, Ct-HBx downregulates the expression of TXNIP (thioredoxin-interacting protein), a tumor suppressor that normally inhibits glycolysis. With TXNIP suppressed, key glycolytic enzymes such as lactate dehydrogenase A (LDHA) are upregulated, driving increased glucose uptake and lactate production, while enzymes involved in mitochondrial metabolism, including PDH, MDH2, CS, IDH1, are downregulated. This reprogramming enhances glycolytic activity and reduces ATP production from oxidative phosphorylation (Zhang et al. 2021). The Warburg effect not only meets the biosynthetic and energy demands of rapidly growing tumor cells but also creates a tumor microenvironment that favors metastasis and immune evasion (Wang et al. 2025). In addition, certain HBx variants, such as HBx-K130M/V131I, can reprogram host cellular metabolism in ways that favor both HBV replication and tumor development. A study showed that it promotes activation of the PI3K/AKT pathway, reduces the expression of forkhead box O1 (FOXO1), and supports cell survival and proliferation (Chiu et al. 2019). These effects include metabolic shifts consistent with increased nutrient acquisition and anabolic processes, which are advantageous both for viral replication and for the cancer phenotype.

## Natural products and their molecular mechanisms targeting HBx and HBx-driven pathways in HBV infection and hepatocellular carcinoma

**Figure 5.**
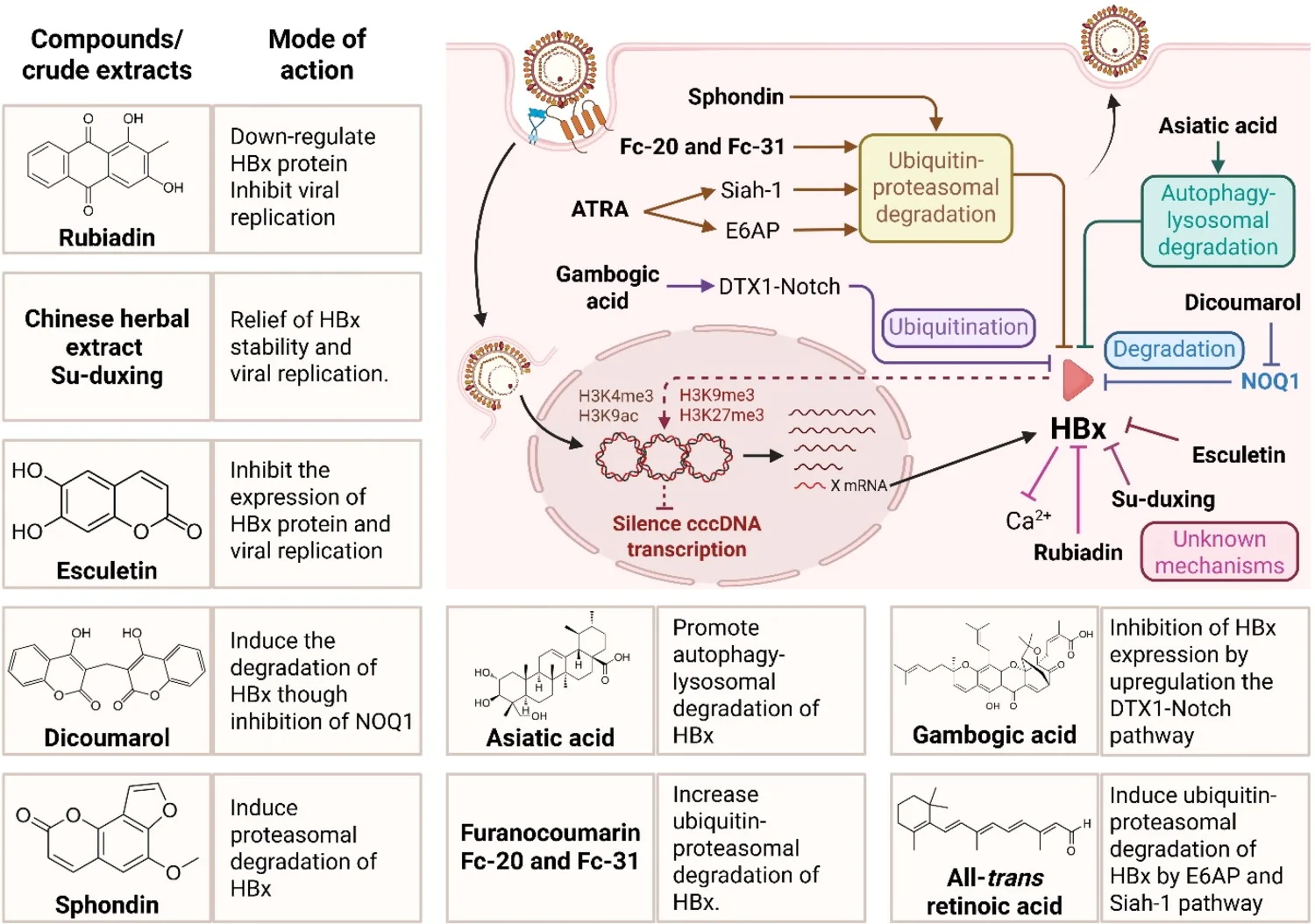
The inhibitory effect of natural products and compounds on HBx in HBV infection and their molecular mechanisms. (Create by BioRender.com/ Mahidol University).

**Figure 6.**
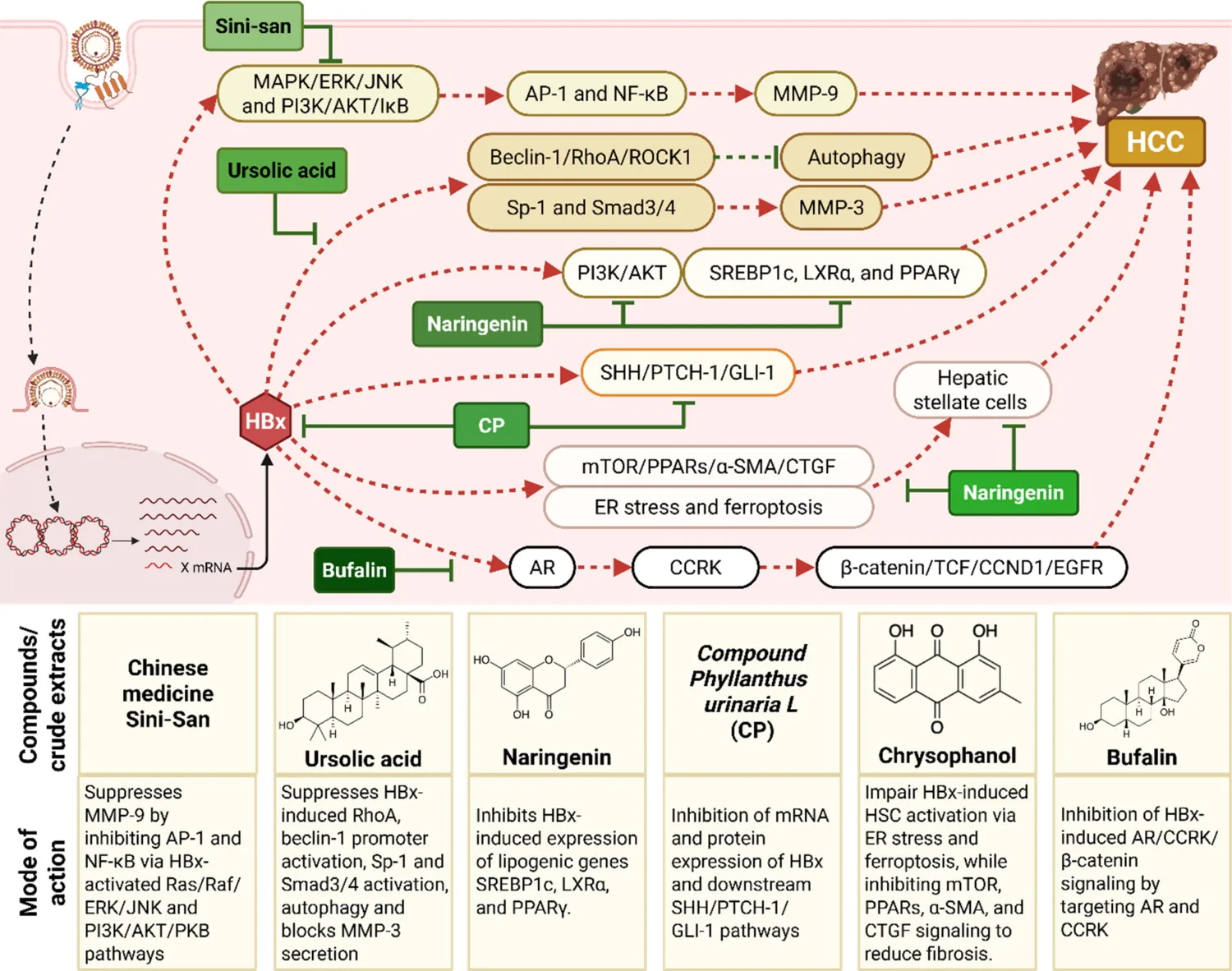
The inhibitory effect of natural products and compounds on HBx-modulated HCC development and their molecular mechanisms. (Create by BioRender.com / Mahidol University).

Natural compounds and products derived from plants and animals in some cases show significant potential in targeting HBx-induced HCC and HBV replication through diverse molecular mechanisms. HBx is a viral regulatory protein that promotes hepatocarcinogenesis by interacting with host signaling pathways and non-coding RNAs, while phytochemicals have been shown to counteract these effects *via* antiviral, pro-apoptotic, and cell cycle-regulating activities. The natural compounds and products reviewed are divided into two mechanistically distinct categories. The first includes direct HBx-targeting agents that reduce HBx expression and suppress HBV persistence (Table 1) and the second includes compounds that primarily modulate downstream HBx-associated signaling pathways involved in hepatocarcinogenesis (Table 2). The mechanisms underlying the anti-HBx effects in HBV infection and HCC development of natural products and plant extracts have been depicted Figure 5 and Figure 6.

## Natural products directly targeting HBx to suppress HBV replication and persistence

### Chinese herbal extract Su-duxing

Su-duxing is a Chinese herbal extract that has been studied for its potent inhibitory effects against both wild-type and entecavir-resistant HBV *in vitro* and *in vivo*. The formula is an improved version based on Su-du injecta, which was previously used for treating human immunodeficiency virus (HIV). Su-duxing extract contains 6 active compounds known for their anti-HBV properties, including matrine, oxymatrine, chlorogenic acid, sophocarpine, baicalein, and wogonin, with paired combinations showing greater suppression of HBV DNA and HBsAg than individual constituents, consistent with mechanistic synergy (Liu et al. 2018). Su-duxing demonstrates reproducible anti‑HBV activity across wild‑type (HepG2.2.15) and entecavir‑resistant (HepG2.A64) systems and in an AAV8‑HBV (1.3‑mer) mouse model. Moreover, the combination between Su-duxing and entecavir outperformed either agent alone, supporting complementary mechanisms and a higher barrier to resistance. Transcriptomics and KEGG mapping implicated destabilization of HBx and interference with viral replication, deregulation of early cell‑cycle checkpoints, and type‑I interferon induction; qRT‑PCR confirmed Su-duxing had significant alterations in the expression of 4 genes: CCNA2 and CDKN1A/p21, ATF4, FAS, nominating a coherent network that can attenuate HBV transcription/replication and antigen production (Liu et al. 2018). Despite these promising data, Su-duxing remains a complex multi‑component extract with unknown recipe, lacking direct evidence of effects on cccDNA or biophysical interaction with HBx. Further studies are required to identify the specific bioactive constituents responsible for these effects and to clarify their precise molecular targets.

### Rubiadin

Rubiadin (1,3-Dihydroxy-2-methylanthracene-9,10-dione) is a bioactive anthraquinone isolated from Rubiaceae family plants like *Prismatomeris connata* and *Rubia cordifolia*, which have been studied for hepatoprotective effects and the treatment of hepatitis, hepatic fibrosis, and silicosis (Rao et al. 2006; Peng et al. 2017). Rubiadin, directly extracted and purified from *Prismatomeris connata*, can significantly reduce the secretion of viral antigen HBsAg, HBeAg, and HBcAg in HBV-infected hepatocytes. The compound also lowers HBV DNA levels in HepG2.2.15 cells with efficacy comparable to lamivudine (3TC), a standard antiviral drug (Peng et al. 2017). Importantly, rubiadin treatment also decreases the expression of the HBx protein and significantly decreases cytosolic free Ca^2+^ concentration in HepG2.2.15 cells (Peng et al. 2017), suggesting that modulation of calcium homeostasis through HBx is an important component of its antiviral mechanism. Mechanistically, HBx is known to regulate intracellular Ca^2+^ homeostasis by enhancing store-operated calcium entry (SOCE) at the plasma membrane (Yang and Bouchard 2012), influencing mitochondrial Ca^2+^ uptake (Kong et al. 2021), further increasing cytosolic Ca^2+^ levels and sustaining Pyk2/FAK/Src/downstream MAPK/NF‑κB/AP‑1 cascades required for viral replication (Bouchard et al. 2006). Rubiadin represents a promising candidate for HBx-targeted antiviral therapy because it appears to suppress HBV replication by modulating host calcium (Ca^2+^) signaling pathways that are essential for HBx function. However, current evidence is largely derived from *in vitro* hepatoma cell models, and the precise molecular targets of rubiadin within the Ca^2+^ regulatory network remain unclear.

### Coumarin derivatives

*Esculetin*, also known as 6,7-dihydroxycoumarin, is a hydroxycoumarin compound classified under benzopyrones, and is naturally found in several plants, including *Sonchus grandifolius*, *Microsorium fortunei*, and *Aesculus turbinata* (Information NCfB 2005; Garg et al. 2022; Hossain et al. 2023). Esculetin is reported to effectively reduce inflammation by inhibiting key pathways such as NF-κB and MAPK, and has demonstrated the ability to inhibit tumor cell proliferation and induce apoptosis across multiple cancer types, including colorectal, gastric, and hepatocellular carcinomas by modulating signaling pathways such as Wnt/β-catenin and PI3K/Akt, which are essential in cancer development (Fan et al. 2017; Li et al. 2017; Liu et al., 2024). In HepG2.2.15 cells, purified esculetin from *Microsorium fortunei* significantly reduced HBV DNA and viral antigens, downregulated HBx protein in a dose‑dependent manner, and its inhibitory effect was stronger compared to 3TC. Translational relevance was supported in the duck hepatitis B virus (DHBV) model, where esculetin treatment significantly lowered DHBV DNA, DHBsAg, and DHBeAg, and improved ALT/AST, indicating both antiviral and hepatoprotective effects *in vivo* (Huang et al. 2019). Additional esculetin derivatives bearing 7‑position amino‑containing side chains or C‑4/C‑8 substitutions showed selective HBeAg or HBsAg inhibition, with improved metabolic stability, offering preliminary SAR that positions the 6,7‑dihydroxy pharmacophore as a tractable scaffold for HBx/antigen‑lowering leads (Ye et al. 2023). Despite compelling evidence that esculetin downregulates HBx protein in a dose‑dependent manner, the available studies provide no mechanistic resolution of how esculetin alters HBx stability, and direct effects on cccDNA transcriptional activity, a major HBx‑dependent regulatory node, have not been examined, leaving a gap between HBx suppression and the extent of downstream antiviral benefit.

*Dicoumarol*, a 3,3′-methylenebis-(4-hydroxycoumarin), is a natural bis‑coumarin which was first discovered from moldy alfalfa (Melilotus) by Karl Paul Link in 1940 and can now be synthesized from p-nitrobenzene ketone and 4-hydroxycoumarin (Information NCfB, 2025). Dicoumarol is a vitamin‑K antagonist that inhibits VKORC1, and it is also a competitive inhibitor of NAD(P)H:quinone oxidoreductase‑1 (NQO1) by competing with NAD(P)H at the FAD site. Therefore, it is best known as an oral anticoagulant and historically served as a precursor for the development of warfarin (Sun et al. 2020; Silva et al. 2022; Information NCfB, 2025). Recently, dicoumarol has been studied for its anticancer and antiviral properties, though its effects on HBV are not extensively documented (Silva et al. 2022). Dicoumarol exhibits direct anti‑HBV effects through an HBx‑centered mechanism: in HBV‑infected hepatocytes and *in vivo*, dicoumarol promotes HBx degradation by inhibiting NQO1’s gatekeeper function on the 20S proteasome, thereby reducing HBx recruitment to cccDNA, collapsing cccDNA transcriptional activity, without altering cccDNA copy number, and lowering HBsAg, HBV DNA, and core proteins (Cheng et al. 2021). In HCC, NQO1 antagonism of dicoumarol intersects tumor biology by sensitizing HCC cells to ferroptosis. Systems analysis in HCC identifies NQO1 and SLC7A11 as poor‑prognosis, ferroptosis‑resistance genes; combining dicoumarol with imidazole‑ketone‑erastin enhances ferroptotic death and suppresses HCC xenografts, with increased iron deposition and up‑regulation of ferroptosis markers (Yang et al. 2025). Given HBx’s role in oncogenic signaling and cccDNA transcription, the effects of dicoumarol on HBx depletion plus ferroptosis sensitization provide a coherent rationale for evaluating dicoumarol‑based therapy in HBV‑related HCC. However, current evidence for dicoumarol activity remains largely limited to experimental studies, and its clinical application is constrained by anticoagulant-related toxicity and lack of specificity for viral targets.

### Furanocoumarin derivatives

*Furanocoumarins* are a class of organic compounds consisting of a furan ring fused to a coumarin backbone, forming either linear (psoralen-type) or angular (angelicin-type) scaffolds, and they display characteristic UV‑absorbing, photosensitizing, and lipophilic chemical properties that influence their biological behavior. They are secondary metabolites primarily found in plant families such as Apiaceae, Rutaceae, Moraceae, and Fabaceae. Biopharmaceutically, they exhibit diverse activities, including phototoxic, anticancer, neuroprotective, osteoprotective, antioxidant, and antimicrobial effects (Bruni et al. 2019).

*Sphondin*, 6‑methoxyfuro[2,3‑h]chromen‑2‑one, is a naturally occurring angular furanocoumarin, a type of compound commonly found in the Apiaceae (Umbelliferae) family of plants, such as *Heracleum species* and *Coriandrum sativum* (coriander) (Information NCfB, 2025). Previous pharmacological studies demonstrated that sphondin can inhibit inducible nitric oxide synthase and suppress IL-1β-induced cyclooxygenase-2 (COX-2) expression, suggesting its role in regulating inflammatory signaling pathways (Yang et al. 2002). In HBV-infected hepatocytes and humanized-liver mice, sphondin robustly reduces HBsAg and HBV RNAs while leaving cccDNA copy number unchanged, indicating transcriptional silencing of cccDNA rather than DNA loss. Mechanistically, sphondin binds HBx directly at Arg72 to promote ubiquitin‑dependent, 26S proteasome‑mediated HBx degradation, thereby reducing HBx recruitment to cccDNA and converting the cccDNA minichromosome to a less transcriptionally active state. HBx loss or an HBx‑R72A mutant abolishes the antiviral effect, and the combination of sphondin and entecavir yields synergistic suppression of capsid‑derived DNA in cells and greater HBsAg/HBV DNA declines in humanized mice, supporting rational NA combinations for antigen reduction (Ren et al. 2023). However, sphondin exhibits poor aqueous solubility and a relatively short half-life, which may limit its pharmacokinetic properties and clinical bioavailability (Information NCfB, 2025).

*Fc-20 and Fc-31* are artificial furanocoumarin derivatives that demonstrate potent anti‑HBV and anti‑HBx activity by markedly suppressing HBV 3.5‑kb RNA, cccDNA, virion release, and the secretion of HBsAg and HBeAg, while remaining non‑cytotoxic even at high concentrations. Their antiviral effect is a direct interaction with HBx, triggering its proteasome‑dependent degradation, which in turn impairs HBx‑driven transcription, reduces activation of HBx‑dependent promoters including cyclin D1 and HBx promoters, and diminishes H3K4me3 active histone marks on cccDNA, thereby establishing a repressive epigenetic state that limits HBV transcription and replication (Tyagi et al. 2024). Given that HBx relieves SETDB1-dependent repression and supports active cccDNA chromatin, the loss of H3K4me3 after Fc-20/Fc-31 treatment is mechanistically compatible with restoration of host repressive control, but whether these compounds directly permit SETDB1 or PRMT1 reactivation has not yet been experimentally resolved. Plus, the ability of Fc-20 and Fc-31 to inhibit tenofovir-resistant CYEI mutants and synergize with entecavir suggests combination therapies, especially with other HBx degraders or epigenetic agents that maintain repressive chromatin states on cccDNA, to fully evaluate their potential in achieving functional HBV cure.

### Asiatic acid (AA)

Pentacyclic triterpenoids are crucial natural bioactive substances made up of 30 carbon atoms arranged into five (penta-) ring structures that are widely found in plants and fungi, and are known for their diverse pharmacological activities, including anti-inflammatory, antioxidant, antiviral, and anticancer activities (Li et al. 2023). Asiatic acid is a naturally occurring pentacyclic triterpenoid compound derived mostly from *Centella asiatica* (Gotu Kola), a medicinal herb used in Ayurvedic and traditional Asian medicine (Information NCfB, 2025). AA exhibits multi‑pathway anticancer activity by inhibiting proliferation, inducing apoptosis, regulating autophagy, suppressing metastasis *via* PI3K/AKT, ERK/p38 MAPK, Wnt/β‑catenin, and reversing chemoresistance (Chen et al. 2025; Liang et al. 2025). In HBV infection, AA exhibits potent anti-HBV activity *in vitro* and *in vivo* by markedly reducing HBV RNAs, HBV core DNA, and viral antigens, acting primarily as a transcriptional repressor rather than a cccDNA-eliminating agent. AA facilitates the degradation of HBx through the autophagy-lysosomal pathway, which then reduces HBx binding to cccDNA and shifts histone marks toward a repressive state by reducing histone modification H3K4me3 and enrichment of transcriptional repressive markers, H3K9me3 and H3K27me3 on cccDNA microchromosomes, thereby epigenetically silencing cccDNA transcription (Li et al. 2024). These findings are consistent with a model in which HBx removal permits reestablishment of host repressive machinery, including EZH2/PRC2-associated H3K27 trimethylation and SETDB1-associated H3K9 trimethylation, although direct measurement of EZH2 or SETDB1 recruitment after asiatic acid treatment has not yet been reported (Rivière et al. 2015; Salerno et al. 2020). Future research should evaluate combination therapy with nucleos(t)ide analogues or agents that enhance repressive chromatin regulation, particularly pathways associated with EZH2- or SETDB1-mediated histone methylation, which can therefore promote more durable silencing of the HBV transcriptional reservoir and enhance anti-HBV activity.

### Gambogic acid (GA)

Gambogic acid is a xanthonoid compound derived from the resin of *Garcinia hanburyi* with a wide range of biological activities, primarily recognized for its anticancer effects (Hatami et al. 2020). GA exhibits broad biopharmaceutical activity by inducing apoptosis, autophagy, cell-cycle arrest, and suppressing invasion, metastasis, and chemoresistance through diverse modulation of pathways including p53/MDM2, Bcl‑2/caspase, ROS–JNK/p38, NF‑κB, PI3K/AKT/mTOR, STAT3, and HIF‑1α/VEGF (Hatami et al. 2020; Liu et al. 2020). In recent years, GA was identified as a potential inhibitor of HBV replication through an HBx-associated mechanism. In HBV-infected HepG2-NTCP cells, GA reduced HBx expression at both mRNA and protein levels and caused dose-dependent decreases in extracellular HBV RNAs, HBV DNA, HBsAg, HBeAg, and HBc protein without significant cytotoxicity. RNA sequencing analysis revealed that GA upregulated the expression of Deltex1 (DTX1), an E3 ubiquitin ligase, and enhanced Notch signaling, and the study concluded that GA-mediated inhibition of HBV replication occurs through activation of the DTX1-Notch signaling pathway (Wen et al. 2024). However, the current evidence supports an association between GA treatment, DTX1 upregulation, and Notch-pathway activation, but it does not yet demonstrate that DTX1 directly ubiquitinates HBx, recruits HBx to the proteasome, or alters HBx stability. Therefore, a visible gap in understanding how GA influences cccDNA regulation at the chromatin level and pharmacokinetic constraint related to GA’s poor aqueous solubility limits the clinical development of GA as an antiviral agent.

### All-trans retinoic acid (ATRA)

All-trans retinoic acid (ATRA), also known as tretinoin, is a biologically active metabolite of vitamin A with significant roles in development, cell differentiation, and immune function (Information NCfB, 2025). In regard to antiviral activities, ATRA has demonstrated inhibitory effects on HBV replication by promoting the degradation of the HBx protein through two distinct, p53-dependent mechanisms. Firstly, ATRA can up-regulate p53 levels, which in turn increases the expression of seven in absentia homolog 1 (Siah-1), an E3 ubiquitin ligase. Subsequently, Siah-1 induces the ubiquitination and proteasomal degradation of HBx, reducing its intracellular levels and inhibiting HBV replication. This process is p53-dependent, as knockdown of p53 abolishes the effect of ATRA on HBx degradation and HBV replication (Han and Jang 2023). Additionally, ATRA induces promoter hypomethylation of the E6-associated protein (E6AP) gene. Elevated E6AP levels enhance its interaction with HBx, leading to the ubiquitination and subsequent proteasomal degradation of HBx, providing another pathway for reducing HBx levels and suppressing HBV replication (Han and Jang 2024). ATRA also promotes cellular senescence by upregulating tumor suppressor genes like p16 and p21, which can further inhibit HBV replication by limiting host cell proliferation (Park et al. 2011).

## Natural products modulating HBx‑driven pathways in hepatocellular carcinogenesis

### Chinese medicine Sini-San (SNS)

Sini-San (SNS) is a traditional Chinese medicinal formula composed of four main herbs: Bupleurum (*Radix Bupleuri*), white peony (*Radix Paeoniae Alb*a), immature bitter orange (*Fructus Aurantii Immaturus*), and licorice (*Radix Glycyrrhizae*). SNS is traditionally used to soothe the liver, regulate Qi, and harmonize spleen–liver dysfunction. It contains more than 338 identified compounds, with paeoniflorin, naringin, glycyrrhizic acid, and saikosaponin A serving as major bioactive and quality-marker constituents. Modern studies show that SNS exhibits hepatoprotective, anti-inflammatory, antioxidant, and anticancer activities, supported by diverse mechanistic pathways including PI3K/AKT, MAPK, NF‑κB, Nrf2/HO‑1, AMPK/SIRT1, and PPAR‑γ signaling (Li et al. 2026). *In vitro* studies utilizing the HepG2 cell line with inducible HBx expression revealed that SNS treatment significantly reduced HBx-induced cell migration and invasion. This effect is attributed to the downregulation of matrix metalloproteinase-9 (MMP-9) expression and activity, a key enzyme involved in extracellular matrix degradation and tumor metastasis. Specifically, SNS blocks HBx-mediated activation of AP‑1 and NF‑κB, and upstream MAPK (ERK, JNK, p38) and PI3K/AKT/IκB phosphorylation and subsequent MMP-9 expression (Lin et al. 2015). Importantly, several SNS constituents including saikosaponins, naringin, glycyrrhizic acid, and isorhamnetin, individually modulate PI3K/AKT, NF‑κB, AP‑1, and oxidative stress pathways (Li et al. 2026), suggesting that SNS’s anti‑HBx effects emerge from synergistic multi‑component actions. However, current data provide no evidence that SNS or its components directly bind HBx, degrade HBx, or modulate HBx–cccDNA interactions; instead, SNS predominantly attenuates downstream HBx‑triggered signaling, representing an indirect but biologically meaningful antagonism.

### Compound Phyllanthus urinaria L (CP)

Compound *Phyllanthus urinaria L.* (CP) is a traditional Chinese medicine formula that has been widely used in clinical practice for treating hepatocellular carcinoma (HCC), especially HBV-related HCC, due to its antiviral and antifibrotic properties. CP formula comprises *Phyllanthus urinaria L, Astragalus membranaceus (Fisch.) Bunge, Scutellaria barbata D. Don, Curcuma zedoaria (Christm.) Rosc, and Cremastra appendiculata (D. Don) Makino* (Jung et al. 2015). CP is believed to inhibit HBV-related HCC by inactivating the HBx-Sonic Hedgehog (SHH) pathway axis. Mechanistic investigations indicated that HBx activates the SHH signaling pathway by upregulating components such as SHH, PTCH-1, SMO, GLI-1, and GLI-2, which are associated with tumorigenesis, while CP treatment effectively inactivates the SHH pathway and mitigating HBx-induced HCC progression (Li et al. 2019). Further research is needed to fully elucidate CP mechanisms and potential clinical applications in treating this condition.

### Ursolic acid (UA)

Ursolic acid (UA) is a natural pentacyclic triterpenoid compound commonly found in fruits, especially apple peels, bilberries, and some popular herbs like rosemary, thyme, oregano, basil, and medicinal plants such as *Mirabilis jalapa* (Information NCfB, 2004; Seo et al. 2018). UA exhibits a wide range of pharmacological effects, from anti-inflammatory effects by suppressing inflammatory pathways like NF-κB signaling to anticancer activity through inducing apoptosis in cancer cells by targeting mitochondrial pathways and inhibiting proliferation (Panda et al. 2022). UA is also reported to have hepatoprotective properties against liver fibrosis and enhance liver regeneration (Seo et al. 2018). UA exerts potent anti‑HCC activity, particularly in the context of HBV‑related carcinogenesis. Ursolic acid interferes with HBx-regulated autophagy signaling rather than simply increasing autophagy. HBx promotes a pro-survival form of autophagy through RhoA-ROCK1-dependent activation of Beclin-1 transcription, which contributes to chemoresistance in hepatoma cells. Ursolic acid disrupts this HBx-mediated pathway by inhibiting RhoA activation and suppressing Beclin-1 promoter activity, thereby restoring autophagic flux toward a cytotoxic outcome. As a result, UA reverses HBx-induced resistance to anticancer drugs and attenuates HBx-driven cell survival and migration (Chang et al. 2016). In addition, UA suppresses HBx-mediated transcriptional programs involving Sp1 and Smad3/4, leading to reduced MMP3 secretion and inhibition of invasive behavior. UA also restores TGF-β-induced apoptosis, a mechanism that contributes to tumor survival, in HBx-expressing Hep3B cells, reversing HBx’s anti-apoptotic action (Wu et al. 2011).

### Naringenin (nar)

Naringenin (Nar) is a naturally occurring flavanone, a dihydroflavonoid with hydroxy groups at positions 5, 7, and 4′, making it a trihydroxyflavanone. It is primarily found in citrus fruits, particularly in grapefruits and oranges, as well as in other plants like cherry buds and sumac fruits, and is known for its diverse biological activities, including antioxidant, anti-inflammatory, and anticancer properties (Information NCfB, 2004; Motallebi et al. 2022). In the context of HBx-targeting, Nar treatment in HBx-transgenic mice significantly reduced hepatic lipid deposits, ballooning hepatocytes, and abnormal sinusoid arrangements. Nar decreased HBx-induced expression of sterol regulatory element-binding protein 1c (SREBP1c) and liver X receptor alpha (LXRα), key regulators of fatty acid synthase (FAS) and acetyl-CoA carboxylase, and peroxisome proliferator-activated receptor gamma (PPARγ) and CD36, which are drivers of adipogenesis and lipid uptake. Furthermore, HBx enhances PI3K/Akt activity, promoting lipogenesis and resistance to apoptosis, while Nar suppresses phosphorylation of PI3K and Akt, disrupting this pro-lipogenic signaling. Plus, HBx upregulates CCAAT/enhancer-binding protein alpha (C/EBPα), which activates PPARγ to drive steatosis, whereas Nar reduces C/EBPα protein levels, breaking the link between HBx and PPARγ-driven lipid accumulation (Lin et al. 2017). In diet‑induced fatty liver models, naringenin markedly suppresses NF‑κB p65 activation, leading to down‑regulation of the NLRP3 inflammasome, reduced maturation of IL‑1β and IL‑18 (Wang et al. 2020). These data strongly suggest that naringenin indirectly antagonizes HBx oncogenic signaling by disrupting the NF‑κB–NLRP3 inflammatory pathway.

### Chrysophanol

Chrysophanol, also known as chrysophanic acid, is a naturally occurring anthraquinone compound and is commonly found in various plants, including *Rheum palmatum* (rhubarb), *Aloe vera*, and certain species of docks and sorrels. It is characterized by its chemical structure as a trihydroxyanthraquinone with a methyl substituent at the C-3 position, making it a derivative of chrysazin (Information NCfB, 2004). Chrysophanol possesses various pharmacological effects and exhibits anticancer properties by inducing necrosis in human liver cancer cells, making it a therapeutic candidate for HCC (Su et al. 2020). Additionally, chrysophanol impairs HBx-induced activation of hepatic stellate cells (HSCs) through mechanisms involving endoplasmic reticulum (ER) stress and ferroptosis-dependent pathways, a form of regulated cell death characterized by iron-dependent lipid peroxidation, through increased expression of ER stress markers, including Bip, CHOP, and phosphorylated IRE1α. Hence, there was an accumulation of lipid reactive oxygen species (ROS), indicating elevated oxidative stress within the cells (Kuo et al. 2020). Another crucial mechanism is that chrysophanol has been shown to inhibit the mammalian target of rapamycin (mTOR) and peroxisome proliferator-activated receptors (PPARs) signaling pathways, which are upregulated by HBx in HSCs. This inhibition helps to reduce HBx-induced liver fibrosis by downregulating fibrogenic markers such as α-smooth muscle actin (α-SMA) and connective tissue growth factor (CTGF) (Lin et al. 2024). Consequently, these mechanisms collectively contribute to reducing liver fibrosis and potentially mitigating the progression of HCC associated with HBx expression, supporting chrysophanol as a potential therapeutic candidate.

### Bufalin

Bufalin is a cardiotonic steroid isolated from the skin and parotid glands of the Chinese toad, *Bufo gargarizans*, and other toad species. Bufalin is toxic and classified as a danger substance due to its cardiotoxic effects. However, bufalin exhibits antitumor activity against various cancer cell lines, including hepatocellular carcinoma and lung cancer (Information NCfB, 2004). Bufalin acts as a potential anti-HCC therapeutic candidate by blocking HBx-induced signaling pathways. Specifically, it induces dephosphorylation of androgen receptor (AR), reducing its activity, and promoting the degradation of cell cycle-related kinase (CCRK). This dual action resulted in the suppression of the β-catenin/TCF signaling pathway, leading to decreased expression of downstream targets such as cyclin D1 (CCND1) and epidermal growth factor receptor (EGFR), both of which are associated with the oncogenic effects of HBx, reducing the cell proliferation and tumor progression (Wang et al. 2014; Yu et al. 2020).

## Discussion

Natural products have gained attention as therapeutic agents in HBV‑related HCC through both direct targeting of HBx and indirect suppression of HBx‑driven oncogenic pathways. Natural products and traditional medicines provide structurally diverse agents capable of modulating these HBx-driven processes at multiple levels. However, exploration of their anti-HBx and anti-HCC effects must be balanced against careful evaluation of safety, pharmacokinetics, and translational feasibility. This discussion synthesizes available evidence for representative HBV-related and HCC-related natural agents, focusing on toxicology, systemic exposure, and clinical applications, which are summarized in Table 3.

### Safety and toxicological evidence

Safety profiles vary markedly across HBV‑ and HCC‑related compounds. Naringenin (Rebello et al. 2020), ursolic acid (Wang et al. 2013), asiatic acid (Grimaldi et al. 1990), esculetin (Zhang et al. 2022), and sphondin demonstrate the most favorable safety profiles, supported by repeated animal studies and long-term dietary or herbal exposure without overt organ toxicity at experimental doses (typically 20 − 200 mg/kg/day orally). Sini‑San and Compound *Phyllanthus urinaria L.* are widely used in East Asia for chronic liver disorders and modern toxicology studies confirm hepatoprotection with no significant renal or hematological toxicity (Jiang et al. 2003; Liu et al., 2024; Li et al. 2026). Based on the double‑blinded, placebo‑controlled clinical trial by Chan *et al.* (2003), *Phyllanthus urinaris L.* has direct human translation evidence in chronic HBV infection with oral administration of *P. urinaris* at 1-3 g three times daily for 6 months was well tolerated with no serious adverse events, but did not result in significant reduction of HBV DNA, HBeAg secretion, indicating limited standalone antiviral efficacy despite clinical safety (Chan et al. 2003).

In contrast, gambogic acid and bufalin show narrow safety margins. Gambogic acid displays strong cytotoxicity but also demonstrates dose-dependent systemic toxicity, with the liver and kidney identified as major toxicity targets in chronic animal studies, requiring controlled dosing or delivery systems (Guo et al. 2006; Xu et al. 2025). Bufalin is associated with severe cardiotoxicity linked to Na^+^/K^+^‑ATPase inhibition, leading to dose-dependent cardiotoxicity, including arrhythmias, conduction block, and sudden cardiac death in animal models and humans, which makes chronic administration challenging, particularly in patients with underlying liver disease who may require long-term treatment (Botelho et al. 2019). Dicoumarol carries bleeding risk due to anticoagulant activity through vitamin K epoxide reductase inhibition with historical clinical use documenting hemorrhagic complications, especially with prolonged exposure or overdose, leading to its replacement by warfarin in routine practice (Sun et al. 2020). ATRA has dose-limiting toxicities from extensive clinical use, including mucocutaneous toxicity, headache, hypertriglyceridemia, and elevated liver enzymes, while more serious complications include retinoic acid syndrome (differentiation syndrome) and hepatotoxicity, reported in approximately 20-30% of patients as reversible transaminase elevations (Roenigk Jr 1988; Woods and Norsworthy 2023).

Comprehensive reviews report that repeated oral administration of chrysophanol in rodents (typically 20–100 mg/kg/day) leads to elevated serum transaminases, histopathological liver injury, and renal tubular damage, indicating hepatotoxicity and nephrotoxicity at pharmacologically relevant doses (Xie et al. 2019). For rubiadin, Su‑duxing, and furanocoumarins (Fc‑20, Fc‑31), available toxicological data remain limited, requiring cautious interpretation and further study.

### Pharmacokinetics, bioavailability, and translation evidence

Poor bioavailability is a common limitation among natural products. Among the compounds reviewed, Naringenin currently has the strongest dose-translation support. In HBx-related steatosis models, naringenin produced biological effects at 25–100 μM, whereas a randomized clinical pharmacokinetic study reported no relevant adverse events in healthy adults after oral doses of 150–900 mg, with peak serum concentrations reaching 15.76 ± 7.88 μM at 150 mg and 48.45 ± 7.88 μM at 600 mg in healthy volunteers, suggesting that at least the lower end of the experimentally active concentration range may be clinically achievable (Rebello et al. 2020). ATRA has fully characterized human pharmacokinetics and established dosing regimens but is constrained by toxicity and resistance in solid tumors. ATRA is administered orally and shows rapid gastrointestinal absorption, reaching peak plasma concentrations within 1-2 h (Adamson 1994).

For pentacyclic triterpenoids such as asiatic acid and ursolic acid, the dominant problem is poor water solubility, which restricts dissolution and systemic absorption after oral administration (Grimaldi et al. 1990). The absolute oral bioavailability of asiatic acid has been reported to be approximately 16.25% in rats (Yuan et al. 2015), prompting the development of asiatic acid-loaded solid lipid nanoparticles and other lipid-based delivery systems to improve systemic exposure (Dong et al. 2025). Plus, available human pharmacokinetic studies have primarily evaluated systemic exposure following administration of *Centella asiatica* triterpene mixtures rather than purified asiatic acid (Songvut et al. 2019), making it difficult to determine whether exposure levels associated with antiviral efficacy can be reliably achieved in humans. Regarding ursolic acid, the translational field of intravenous lipid-based formulations has undergone early clinical evaluation. In healthy volunteers and patients with advanced solid tumors, both intravenous ursolic acid liposomes and ursolic acid nanoliposomes showed manageable toxicity profiles and approximately linear pharmacokinetics over the tested dose ranges (Wang et al. 2013; Zhu et al. 2013). Although these formulations achieved measurable systemic exposure in clinical studies, it remains unclear whether plasma concentrations equivalent to those associated with anti-HCC activity in experimental models can be consistently achieved and maintained in humans. Gambogic acid presents an even more difficult translational profile with poor water solubility and low bioavailability while also noting poor stability and severe adverse effects. Many studies have reported gambogic acid-layered double hydroxide/liposome nanocomposites to provide sustained release and lower hemolysis than the non-liposomal hybrid; however, such systems remain preclinical (Hatami et al. 2020; Xu et al. 2025).

Su-duxing, rubiadin, esculetin, sphondin, and Fc-20/Fc-31 currently present the greatest dose-translation uncertainty because of either limited pharmacokinetic characterization or no human PK data for these agents. Multi‑component formulations such as Sini‑San and *Compound Phyllanthus urinaria* L. do not follow classical PK models due to their sophisticated remedies, making them difficult to define in single-analyte terms (Zhu et al. 2025). Dicoumarol and all-trans retinoic acid are more advanced pharmacologically because human exposure is established, but both face clear therapeutic-window limitations, including anticoagulant liability for dicoumarol and dose-limiting toxicity for retinoids in liver disease (Adamson 1994; Sun et al. 2020). However, *in vivo* treatment with dicoumarol at doses of 10 mg/kg and 20 mg/kg for 21 days effectively suppressed HBV without observable signs of toxicity, suggesting that antiviral activity can be achieved within a tolerable exposure range (Cheng et al. 2021).

### Experimental model considerations

The interpretation of anti-HBV activity must consider the model system used. Available HBV cell culture models differ substantially in biological relevance and experimental scope, and each system has distinct advantages and limitations. Stable HBV-producing lines such as HepG2.2.15 and inducible replication systems such as HepAD38 are suitable for examining viral gene expression and replication after the viral template is already present, but they do not reproduce natural viral entry (Guo et al. 2023). NTCP-reconstituted systems such as HepG2-NTCP and differentiated Huh7-hNTCP cells support de novo HBV infection and are therefore more informative for compounds affecting authentic infection steps, although infection efficiency depends on receptor expression, clone selection, and culture conditions (Zahoor et al. 2022; Guo et al. 2023). Recently, differentiated immortalized human hepatocytes (imHCs) have also been developed as an infection-competent hepatocyte model that supports HBV infection and replication while retaining key characteristics of differentiated hepatocytes, providing an additional platform for investigating HBV replication and HBx-associated biology (Sa-Ngiamsuntorn et al. 2019; Thongsri et al. 2021). HBx-overexpression models in Huh7, Hep3B, or related hepatoma cells are valuable for exploring HBx-associated signaling, migration, and apoptosis resistance, but these systems cannot alone distinguish that a compound reverses HBx-driven hepatocarcinogenesis rather than exerting general cytotoxic or antiproliferative effects (Slagle et al. 2015). Similarly, integration-dominant models such as PLC/PRF/5 or Hep3B are informative for studying integrated-HBV biology or HBsAg expression, but they should not be interpreted as equivalent to cccDNA-dependent chronic infection (Daemer et al. 1980; Qiu et al. 2015). Therefore, mechanistic conclusions should be matched to the biological context of the model employed and, whenever possible, confirmed across complementary experimental systems, including HBx-specific, infection-competent, and appropriate non-HBx control models.

### Therapeutic implications of C-terminal truncated HBx variants

A major unresolved issue is whether the natural products reviewed here remain effective against C-terminal truncated HBx (Ct-HBx) isoforms that arise after HBV DNA integration. Ct-HBx variants are clinically important because HBV integration breakpoints cluster near the 3′ end of the X gene, generating truncated HBx proteins that promote hepatocarcinogenesis (Zhang et al. 2021). Ct-HBx variants present an emerging therapeutic challenge because compounds that require C-terminal HBx binding may not remain effective against truncated isoforms. This concern is particularly relevant for phytochemicals that induce HBx degradation, because their activity may depend on direct HBx binding and subsequent proteasomal or lysosomal degradation (Scientific TF. 2026). Although furanocoumarins Fc-20 and Fc-31 promote HBx degradation, docking data identified interactions with retained N-terminal HBx residues including Ala3, Arg26, and Lys140, suggesting that these compounds may retain activity against clinically relevant Ct-HBx variants (Tyagi et al. 2024). Nevertheless, direct experimental validation of these compounds in clinically relevant Ct-HBx-expressing models remains unavailable. Furthermore, compounds that modulate downstream Ct-HBx-related pathways may offer an alternative strategy. For example, maslinic acid has been shown to regulate TXNIP expression through an ALKBH5/m6A-dependent mechanism in high glucose-induced endothelial inflammation (Wang et al. 2022), suggesting that phytochemicals may modulate TXNIP-associated metabolic and inflammatory pathways. Similarly, Sini-San and ursolic acid can suppress c-Jun/AP-1-related signaling pathways (Wu et al. 2011; Lin et al. 2015), which have been implicated in Ct-HBx-mediated hepatocarcinogenesis (Sze et al. 2013). However, no clear evidence currently demonstrates that phytochemicals or natural products reverse Ct-HBx oncogenic activity or associated pathways in integrated Ct-HBx-expressing systems such as Huh7- or PLC/PRF/5-based models. Future studies should therefore directly test candidate compounds in models expressing clinically relevant Ct-HBx variants, to bridge this important knowledge gap in natural product-based anti-HCC drug discovery.

### Advantages, limitations and future perspectives

A key advantage of these natural agents and products is their ability to moderate HBx‑related inflammation, metabolism, and survival signaling simultaneously, which match the complex biology of HBV-related liver cancer. Various natural compounds, such as naringenin, ursolic acid, asiatic acid, Sini‑San, and *Phyllanthus urinaria L*., can simultaneously inhibit inflammatory signaling, alter lipid metabolism, and reduce pro‑tumorigenic gene expression, thereby addressing multiple HBx‑associated processes rather than a single target (Nguyen et al. 2021; Motallebi et al. 2022; Dong et al. 2025). This broad activity, together with generally favorable safety profiles, makes these agents particularly attractive for long‑term prevention, early intervention, or adjunct therapy in HBV‑infected individuals. However, an important limitation that emerges from this review is that most anti‑HCC natural products do not inhibit HBx directly but instead suppress downstream pathways that are activated or modulated by HBx. In contrast, several anti‑HBV compounds and screening hits like furanocoumarins (Fc‑20, Fc‑31), certain anthraquinones, and experimental small molecules, have been reported to directly interfere with HBx stability, transcriptional activity, or protein–protein interactions (Tyagi et al. 2024). This distinction has practical consequences: pathway‑level inhibition may reduce tumor progression but does not eliminate the upstream viral oncogenic driver, potentially allowing residual HBx activity and disease persistence. Moreover, pathway inhibition alone may be less effective in advanced HCC, where HBx‑independent oncogenic mechanisms accumulate. Additional limitations include poor oral bioavailability, especially for hydrophobic triterpenoids and xanthones; incomplete toxicological characterization for many compounds; lack of standardized herbal extracts; and a scarcity of well‑designed clinical trials specifically in HBV‑related HCC populations. Potent agents such as gambogic acid and bufalin further suffer from narrow therapeutic windows and serious safety limitations that restrict clinical feasibility (Botelho et al. 2019; Xu et al. 2025).

Future research should therefore move in several directions. First, greater emphasis should be placed on identifying and optimizing natural or nature‑derived compounds that directly target HBx, including its transcriptional function, post‑translational regulation, or interaction with host factors. Second, combination strategies that pair direct HBx inhibitors (anti‑HBV or anti‑HBx agents) with pathway‑modulating anti‑HCC natural products may provide synergistic benefit by suppressing both the viral driver and its oncogenic consequences. Third, advances in liver‑targeted delivery systems, and improved formulations will be essential to translate promising natural compounds from experimental models to clinical application.

## Conclusions

HBx is a central viral regulator that sustains HBV persistence and drives hepatocellular carcinoma through coordinated effects on cccDNA transcription, genomic instability, immune evasion, and activation of oncogenic signaling pathways. Accordingly, natural products targeting HBx‑associated mechanisms fall into two mechanistically distinct classes: direct HBx‑targeting agents, which reduce HBx expression or stability and suppress HBV replication at its viral root, and indirect modulators of HBx‑driven pathways, which attenuate downstream processes contributing to HCC progression. Direct HBx degraders are therefore most relevant to antiviral or functional‑cure‑oriented strategies, whereas indirect pathway modulators may be better suited for disease modification, chemoprevention, or adjunctive therapy to limit tumor development. Recognizing this mechanistic distinction is essential for rational therapeutic development, and further studies are required to improve compound specificity, bioavailability, and safety, and to validate efficacy in HBV‑relevant *in vivo* systems and clinical studies, while exploring combination strategies that integrate viral suppression with control of HBx-driven oncogenic signaling.

## Supplementary Material

Supplementary Information.docx

## Data Availability

This review is based on curated and extracted data from previously published studies. The compiled review dataset is available from the corresponding author upon reasonable request. To improve transparency and reproducibility, the study-selection and data-extraction dataset used to generate Tables 1 and 2 is provided as Supplementary Table S1.
