# Supplementary material for: Natural products targeting hepatitis B virus X protein and HBx-associated pathways in HBV-related hepatocellular carcinoma: mechanisms and therapeutic potential
Source: Pharm Biol. 2026 Sep 4;64(1):988–1016. doi: 10.1080/13880209.2026.2724161 (PMC13552325; doi:10.1080/13880209.2026.2724161)
Supplement: Supplementary file 1 [file IPHB_A_2724161_SM0875.docx]

**Supplementary Information**

**Supplementary Table S1.** Evidence extraction dataset used for Tables 1-2

| **Compound/ Product** | **Source** | **Model** | **HBx-related endpoint** | **HBV/HCC endpoint** | **Evidence level** | **HBx-targeting classification** |
| --- | --- | --- | --- | --- | --- | --- |
| Su-duxing | Chinese herbal extract | HepG2.2.15, HepG2.A64, C57BL/6 mice | Reduced HBx stability | Decreased HBV replication, HBsAg, HBeAg | *In vitro + In vivo* | Direct |
| Rubiadin | *Prismatomeris connata* | HepG2.2.15 | Reduced HBx expression | Reduced HBV DNA, HBsAg, HBeAg, HBcAg | *In vitro* | Direct |
| Esculetin | *Microsorium fortunei* | HepG2.2.15, ducklings | Reduced HBx expression | Reduced DHBV DNA and viral antigens | *In vitro + In vivo* | Direct |
| Dicoumarol | *Melilotus officinalis* | HepG2-NTCP, HepAD38, PHHs, transgenic mice | HBx degradation; reduced HBx-cccDNA recruitment | Reduced cccDNA transcription and HBV markers | *In vitro + In vivo* | Direct |
| Sphondin | *Heracleum laciniatum* | HepG2, Huh7, HepAD38, mice | Direct HBx binding and degradation | Reduced HBsAg and cccDNA transcription | *In vitro + In vivo* | Direct |
| Asiatic acid | *Centella asiatica* | HepG2-NTCP, HepAD38, Huh7, mice | HBx degradation | Reduced cccDNA transcription | *In vitro + In vivo* | Direct |
| Fc-20/Fc-31 | Synthetic furanocoumarins | HepG2, HepG2.2.15, HepG2-NTCP | HBx degradation | Reduced HBV DNA, cccDNA and viral RNA | *In vitro* | Direct |
| Gambogic acid | *Garcinia hanburyi* | HepG2, Huh7, HepAD38, mice | Reduced HBx expression | Reduced HBV replication markers | *In vitro + In vivo* | Direct |
| ATRA | Vitamin A metabolite | Hep3B, HepG2, NTCP cells | Proteasomal HBx degradation | Reduced HBV replication | *In vitro* | Direct |
| Sini-San | TCM formula | HepG2 | Suppressed HBx-induced signaling | Reduced migration/ invasion | *In vitro* | Indirect |
| Compound *Phyllanthus urinaria* L. | Herbal formula | HepG2, nude mice | Reduced HBx expression | Inhibited SHH pathway and HCC progression | *In vitro + In vivo* | Indirect |
| Ursolic acid | Natural triterpenoid | Huh7, HepG2, Hep3B, Nu/Nu mice | Suppressed HBx-mediated autophagy and survival signaling | Reduced migration, resistance and tumorigenic effects | *In vitro + In vivo* | Indirect |
| Naringenin | Flavanone | HepG2, HBx-Tg mice | Reduced HBx-induced lipogenic signaling | Reduced steatosis-associated HCC phenotypes | *In vitro + In vivo* | Indirect |
| Chrysophanol | *Rheum palmatum* | HSC-T6, LX-2, mice | Inhibited HBx-activated fibrosis pathways | Reduced fibrosis and HSC activation | *In vitro + In vivo* | Indirect |
| Bufalin | *Bufo gargarizans* | HepG2.2.15, PLC5, Huh7, mice | Blocked HBx-induced AR/CCRK signaling | Reduced HCC progression | *In vitro + In vivo* | Indirect |
